# In vitro screening of anti-viral and virucidal effects against SARS-CoV-2 by *Hypericum perforatum* and *Echinacea*

**DOI:** 10.1038/s41598-022-26157-3

**Published:** 2022-12-15

**Authors:** Leena Hussein Bajrai, Sherif Ali El-Kafrawy, Ahmed Mohamed Hassan, Ahmed Majdi Tolah, Rabie Saleh Alnahas, Sayed Sartaj Sohrab, Mohd Rehan, Esam Ibraheem Azhar

**Affiliations:** 1 Special Infectious Agents Unit-BSL3, King Fahd Medical Research Center, King Abdulaziz University, Jeddah, Saudi Arabia; 2 Biochemistry Department, Faculty of Sciences, King Abdulaziz University, Jeddah, Saudi Arabia; 3 Department of Medical Laboratory Sciences, Faculty of Applied Medical Sciences, King Abdulaziz University, Jeddah, Saudi Arabia; 4 Department of Medical Laboratory Technology, Faculty of Applied Medical Sciences, King Abdulaziz University, Rabig, Saudi Arabia; 5 King Fahd Medical Research Center, King Abdulaziz University, Jeddah, Saudi Arabia

**Keywords:** Antivirals, SARS-CoV-2

## Abstract

*Hypericum perforatum* and *Echinacea* are reported to have antiviral activities against several viral infections. In this study, *H. perforatum* (St. John’s Wort) and *Echinacea* were tested in vitro using Vero E6 cells for their anti-viral effects against the newly identified Severe Acute Respiratory Syndrome Coronavirus-2 (SARS-CoV-2) through its infectious cycle from 0 to 48 h post infection. The hypericin of *H. perforatum* and the different parts (roots, seeds, aerial) of two types of *Echinacea* species (*Echinacea purpurea* and *Echinacea angustifolia*) were tested for their anti-viral activities to measure the inhibition of viral load using quantitative real-time polymerase chain reaction (qRT-PCR) on cell culture assay. Interestingly, the *H. perforatum-Echinacea* mixture (1:1 ratio) of *H. perforatum* and *Echinacea* was tested as well on SARS-CoV-2 and showed crucial anti-viral activity competing *H. perforatum* then *Echinacea* effects as anti-viral treatment. Therefore, the results *H. perforatum* and *Echinacea* species, applied in this study showed significant anti-viral and virucidal effects in the following order of potency: *H. perforatum*, *H. perforatum-Echinacea* mixture, and *Echinacea* on SARS-CoV-2 infectious cycle. Additionally, molecular simulation analysis of the compounds with essential proteins (M^pro^ and RdRp) of the SARS-CoV-2 revealed the most potent bioactive compounds such as Echinacin, Echinacoside, Cyanin, Cyanidin 3-(6''-alonylglucoside, Quercetin-3-O-glucuronide, Proanthocyanidins, Rutin, Kaempferol-3-O-rutinoside, and Quercetin-3-O-xyloside. Thus, based on the outcome of this study, it is demanding the setup of clinical trial with specific therapeutic protocol.

## Introduction

Human coronaviruses (HCoVs) continue to pose global threats to human health that started with the spread of severe acute respiratory syndrome coronavirus (SARS-CoV)^1^, followed by the emergence of Middle East respiratory syndrome coronavirus (MERS-CoV) in 2012^2^ and finally the pandemic of severe acute respiratory syndrome coronavirus-2 (SARS-CoV-2) that originated from Wuhan, China. The pandemic caused a global pandemic of COVID-19^3^. With the progression of the disease, intensive production of pro-inflammatory cytokines like: TNF-α, IL-1β, IL-6, IFN-γ, CXCL10, MCP-1, which result in vasculitis, hypercoagulability, and multi-organ damage, leading ultimately to death^4^. Main protease (M^pro^), known as 3-chymotrypsin-like cysteine protease (3CL^pro^), has been considered as an important functional target in the viral life cycle, and therefore as a candidate target for anti-viral drugs against SARS-CoV-2^5,6^ due to its role in the release of functional polypeptides that are encoded by all HCoVs^7,8^. Updated reports about the disease management from COVID-19 have targeted its structure, pathology, and mechanism in order to have the best treatment against the infection^9^. For example, numbers of feasible treatment against SARS-CoV-2 were proposed such as: neuraminidase inhibitors, Remdesivir, Peptide (EK1), abidol, RNA synthesis inhibitors (TDF and 3TC), anti-inflammatory drugs (hormones and other molecules), and Chinese traditional medicine^10^. Furthermore, there were several studies about the synergy between antimalarial drugs, like Chloroquine-Hydroxychloroquine, Remdesivir-Favipiravir for the treatment of COVID-19^11–13^.

The evaluation of herbal remedies and plant extracts that are shown to have an antiviral effect against other coronaviruses might provide an alternative approach to the development of COVID-19 treatments. Several studies have investigated the antiviral activities against other coronaviruses^14^ with high efficacy and low cytotoxicity. For example, glycyrrhizin, an extract from licorice roots, was shown to completely block virus replication^15,16^.

*H. perforatum* (family Hypericaceae), or St. John’s Wort (SJW) has been very well known for a long time as an effective medicinal plant for a range of communicable and non-communicable diseases such as depression, bacterial and viral infections, skin wound, and inflammation^17,18^. *H. perforatum's* metabolites extract from each plant part (roots, seeds and aerial) are different and are chemically defined to naphthodianthrones (hypericin), phloroglucinols (hyperforin), flavonoid glycosides (*hyperoside*), rutin, the flavonoids quercetin and myricetin^19,20^. As an anti-viral agent, *H. perforatum* activities were assessed in vitro and in vivo on infectious bronchitis virus (IBV), Hepatitis C, HIV, and Coronaviruses other than SARS-CoV-2^21,22^. Also, both hyperforin alone and other extracts of *H. perforatum* suppressed cytokine effects in β-cell lines and isolated rat and human pancreatic islets^20,23^. Furthermore, ethyl acetate extraction section of Hypericum (HPE) showed a significant reduction on relative virus titer of IBV in vitro and in vivo, reduction of mRNA expression rate of IL-6, TNF-α, and NF-kB^17^. Based on the potential of *H. perforatum* extract as well as its main polyphenol component hyperforin to counteract the pro‐inflammatory effects of various cytokines, its use was reviewed and proposed to prevent cytokine storm in COVID‐19 patients^24^.

Another medicinal plant, which was applied for many traditional and common remedies like curing cold and flu symptoms and boosting immune system, is *Echinacea*. *Echinacea* is known with nine species of several plants in the genus of *Echinacea,* however, only three of them were used as herbal complements: *E. angustifolia*, *E. purpurea*, and *E. Pallida*. *Echinacea* contains chemical compounds responsible for medicinal properties as: phenols including caffeic acid derivatives and echinacoside, polysaccharides, flavonoids, ketones, and lipophilic alkamides^25,26^. Previous in vitro and in vivo studies, showed that *Echinacea* had an effect on cytokine production^27,28^, increasing the expression of CD69^29^, an impact on natural killer cells^30^, as well as reducing illness severity. In vivo studies showed the anti-inflammatory therapeutic effect on human monocytic THP-1 cells^31^. Also, alkylamides and ketones of *Echinacea* extracts were reported for their anti-inflammatory effects^32–35^. A study in 2009 against H5N1 HPAIV strain showed that the extract of *E. purpurea* interferes with the viral entry into cells by blocking the receptor binding activity of the virus^36^. In another study, the in vitro virucidal and antiviral potential of *Echinacea purpurea* herb and roots ethanolic extract (Echinaforce®) was investigated against human coronaviruses including SARS-CoV-2^37^. The study reported the inactivation of MERS-CoV, SARS-CoV-1, and SARS-CoV-2 using the *Echinacea purpurea* extract.

In continuation to our previous studies to evaluate the antiviral performance of natural antiviral agents against some of the human coronaviruses^16,38,39^, we report in this study, the in vitro anti-viral and virucidal effects of *H. perforatum* (aerial parts) and *Echinacea* species (root, seed, aerial parts) against SARS-CoV-2. We also report a molecular simulation study for the reported bioactive compounds in the selected plants against the potent therapeutic targets, viz. main protein (M^pro^) and RNA dependent polymerase (RdRp) enzymes of SARS-CoV-2.

## Materials and methods

### Cell cultures

Vero E6 cells (ATCC® CRL-1586™) and HEK 293 cells (ATCC® CRL-1573™) were incubated in a 75 cm^2^ cell culture flask containing Dulbecco's modified Eagle's medium (DMEM) supplemented with 10% fetal bovine serum (SIGMA) at 37 °C in a 5% CO_2_ atmosphere. For testing purposes, 96-well plates were seeded with Vero E6 cells at a density of 3 × 10^4^ cells/well and incubated for 24 h at 37 °C with 5% CO_2_ until a confluent monolayer was attained^40^.

### Viral stock

The SARS-CoV-2 isolate used in this study was isolated in the laboratory of BSL-3 from a well-characterized clinical specimen (SARS-CoV-2/human/SAU/85791C/2020, gene bank accession number: MT630432) described earlier^41^. Briefly, isolation was performed in a 75 cm^2^ cell culture flask containing Vero E6 cells in Minimum Essential Media (Gibco, Thermo Fisher)^2^ with 4% of fetal bovine serum and 1% glutamine. Cytopathic effect was monitored daily. In approximately 72 h, nearly complete cell lysis was observed yielding a TCID_50_ of 3.16 × 10^6^ infectious particles per mL. The viral supernatant was used for inoculation in subsequent experiments. All experiments involving live SARS-CoV-2 virus were performed in the biosafety level 3 facility of the Special infectious Agents Unit, King Abdulaziz University.

### *Hypericum perforatum* and *Echinacea* extracts

*H. perforatum* and *Echinacea* products were purchased from gaia HERBS® (Brevard, North Carolina, USA) as gelatin capsules and as liquid; respectively, the products were sold as dietary and herbal supplements. According to the supplier, the whole plant profile is processed in the biocontainment area using water and ethanol to extract the plant's constituents to avoid applying non-ingestible solvents for extraction. Once extracted and filtered, the extract is then concentrated using low heat and low pressure to slowly remove the solvent and preserve the fragile plant constituents. Finally, HPLC analysis is carried to ensure the extract is concentrated to the correct activity levels.

### Sample preparation

The plant materials in concentration of 100 mg/mL^42^ were prepared in a BSL-2 laboratory under reduced light and dissolved in the minimum volume of dimethyl sulfoxide (DMSO, ≥ 99.5%, plant cell culture tested, SIGMA), diluted to the working concentration using culture medium and filtered into 0.22 µm filter^43^. All the extracts were filtered to remove any plant fibers. The mixture of *H. perforatum* and *Echinacea* was prepared by mixing each plant material at 100 mg/mL.

### Cytotoxicity assay

The cytotoxicity assays were performed using the 3-(4,5-dimethylthiazol-2-yl)-2,5-diphenyltetrazolium bromide (MTT, Roche, Germany) protocols as previously reported^44^ with minor modifications. Vero E6 cell monolayers with 4 × 10^3^ cells/mL plated onto 96-well culture plates were washed 3 times with phosphate buffer saline (PBS) 1 ×, pH 7.4. 100 µL of prepared working solution of *H. perforatum*, *Echinacea* and the *H. perforatum-Echinacea* mixture diluted in serum-free DMEM medium (two-fold dilutions, ranging from 0.039 to 5 µg/mL) were added into each well of the 96-well plate and another 100 µL of maintenance medium added onto the wells (3 wells per each dilution in two independent experiments). Control cells were incubated with the corresponding concentrations of DMSO solutions as solvent controls and wells with no additives as negative controls. The cells were incubated at 37 °C for 48 h in a 5% CO_2_ atmosphere, the supernatant was then aspirated, and cells were washed 3 times with PBS, then 20 μL of MTT solution was added to each well and incubated for 4 h at 37 °C. Solubilization solution was added and incubated overnight at 37 °C. The plate was read using an ELISA reader (Synergy 2 microplate, BIOTIK, South Korea) with a reference wavelength of 570 nm (OD570). Cytotoxicity was calculated from mean values according to the following equation: (1 − (OD570 drug/OD570 control) × 100). Cytotoxicity graphs were then generated by plotting percentage of cytotoxicity versus log10 the drug concentration using Graphpad prism 9 (Version 9.0.0) and the CC_50_ was calculated using the nonlinear curve fitting with variable slope where the equation of the fit curve is: Y = 100/(1 + 10^((LogCC50-X)*HillSlope))) where Y is the % cytotoxicity and X is the concentration.

### In vitro micro-inhibition assay

The plant extracts were evaluated as described previously^45^ with some modifications. Briefly, 96-well plates were prepared as mentioned above with Vero E6 cells. Then, the cells were washed twice with PBS, and two-fold serial dilutions of plant materials (*H. perforatum*, *Echinacea* and the *H. perforatum-Echinacea* mixture) (0.316–5 µg/mL) in medium were challenged with a multiplicity of infection (MOI) of 1 of the SARS-CoV-2 isolate and incubated for three days at 37 °C and 5% CO_2_ according to the procedures described in Section “Anti-viral activity assays”. The results were quantified as previously described. The % inhibition was expressed relative to the virus control using dose–response curves. Graphs were then generated by plotting % inhibition versus log10 the drug concentration using Graphpad Prism 9 (Version 9.0.0) and the IC_50_ was calculated using the nonlinear curve fitting with variable slope where the equation of the fit curve is represented by Eq. (1), where Y is the % inhibition and X is the concentration.

1 $$\mathrm{Y}=100/(1+{10}^{\left(LogIC50-X\right)*hillSlope})$$

### Anti-viral activity assays

**Figure 1:**
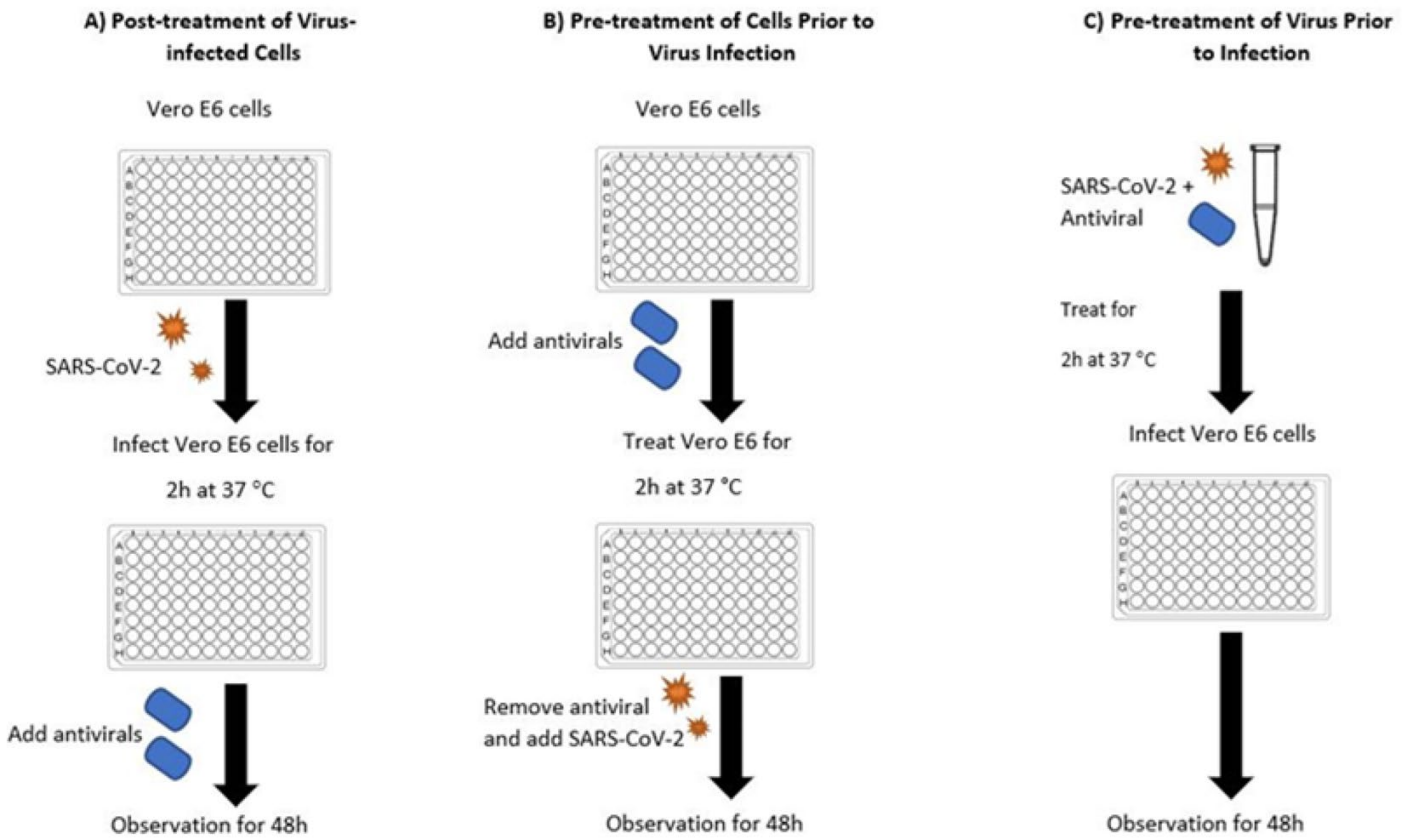
General outline of anti-viral assays. Vero E6 cells were cultured in 96 well-culture plate and used in the three workflows: Post-treatment of Virus-infected Cells (**A**), Pre-treatment of Cells Prior to Virus Infection (**B**), Virucidal (**C**).

The effect of the medicinal plants was tested in the BSL-3 biocontainment laboratory by the anti-viral assays (as represented in Fig. 1). The prepared 96-well plates of Vero E6 were treated with the herbal extracts in triplicate of two independent experiments:

#### Post treatment of virus-infected cells (anti-viral activity)

To determine the effect of *H. perforatum*, *Echinacea* and the *H. perforatum-Echinacea* mixture on SARS CoV-2 infected cells, the Vero E6 cells cultured on 96-well plates were infected with MOI = 1 of the virus isolate for 2 h at 37 °C, as described previously^17^. Then, treated with *H. perforatum*, *Echinacea* and the *H. perforatum-Echinacea* mixture, respectively, at 37 °C for 48 h. The supernatant of the cell samples was collected at 12, 16, 24, 36, and 48 h (3 wells per each time)^46^, the wells of each time point were pooled together to have enough material for RNA extraction and used for qRT-PCR^41^. The percent inhibition of SARS- CoV-2 was evaluated relative to the positive virus control (SARS-CoV-2 on Vero E6 cells with no compounds added).

#### Pre-treatment of cells prior to virus infection (anti-viral activity)

To investigate effect of the medicinal plants on cells to block the cell receptors, the Vero E6 cells cultured on 96-well plates were treated with *H. perforatum*, *Echinacea* and the *H. perforatum-Echinacea* mixture solutions, respectively, at 37 °C for 2 h, and then washed 3 times with PBS solution^17^. The cells were subsequently inoculated with SARS-CoV-2 (MOI = 1) and cultured at 37 °C for 48 h. Supernatant was collected at 12, 16, 24, 36, and 48 h (3 wells per each time point)^46^, pooled together and used for qPCR. The percent inhibition of SARS- CoV-2 was evaluated relative to the positive virus control (SARS-CoV-2 on Vero E6 cells with no compounds added)^41^.

#### Pre-treatment of virus prior to infection (virucidal activity)

To analyze the direct impact of the medicinal plants on SARS-CoV-2 infection, SARS-CoV-2 (MOI = 1) was incubated with *H. perforatum*, *Echinacea* and the *H. perforatum-Echinacea* mixture, respectively, at 37 °C for 2 h^17^. The three extract-treated SARS-CoV-2 were subsequently used to infect Vero E6 cells at 37 °C for 48 h. The percent inhibition of SARS-CoV-2 was evaluated relative to the positive virus control (SARS-CoV-2 on Vero E6 cells with no compounds added) using qRT-PCR^41^ at 12, 16, 24, 36, and 48 h supernatants cell samples^46^. During the post infection cycle, the plates were analyzed by observing virus-induced CPE by light microscope (Nikon-ECLIPSE-Ti, Japan), and was stained crystal violet staining (Sigma-Aldrich, USA), as previously described^44^.

### Quantitative real-time PCR (qRT-PCR)

Viral RNA was extracted from all samples collected directly from the 96 well plates of the anti-viral assay, as previously described^41^ using the QIAmp Viral RNA Mini Kit (Qiagen, Germany) according to the manufacturer’s instructions. Relative quantification of the SARS-CoV-2 viral load was performed by one-step dual-target real time RT-PCR (RealStar SARS-CoV-2 RT-PCR Kit 1.0, Altona Diagnostics, Germany) according to the manufacturer’s instructions using a 7500 Fast Real-Time PCR System (Applied Biosystems, USA). The PCR detects a beta-coronavirus specific target (E-gene), a SARS-CoV- 2 specific target (RdRP-gene) and an internal control. The decrease in viral load was expressed by comparing the cycle threshold values from each sample relative to the Ct values of the pretreatment inoculated sample^47^. The SARS-CoV-2 titers were expressed as PFU equivalents per mL (PEq/mL) using a standard curve generated by testing serial dilutions of the viral stock using qRT-PCR under the same testing conditions as the test samples. Each run included a positive viral template control and no-template negative control. Each sample was tested in duplicate, and the mean is reported as PEq/mL. Y = 100/(1 + 10^((LogIC50-X)*HillSlope)) where Y is the % inhibition and X is the con-centration 4.9.

### Statistical analysis

Data were analyzed with one- or two-way ANOVA with a Tukey’s test for multiple comparisons. *P* < 0.05 and < 0.005 are considered statistically significant. All analyses were per-formed with GraphPad Prism, version 8.

### In silico study

#### Data collection, virtual screening, and re-docking

The in silico study was performed to predict the synergistic effect of *E. angustifolia*, *E. purpurea*, and *H. perforatum* phytochemicals against SARS-CoV-2. Briefly, information about the phytochemical compounds present in the selected plants were searched in published literature and their 3D structures were retrieved from PubChem database in SDF format^48,49^. Moreover, the crystal structures of SARS-CoV-2 Main protease (M^pro^) and (b) of RNA-dependent RNA polymerase (RdRp) proteins were collected from RCSB Protein Databank (http://www.rcsb.org/) with PDB ID:6W63^50^ and PDB ID:7B3C^51^, respectively. Both target structures were prepared using Dockprep tool inbuilt in Chimera program, and all the compounds were prepared in PyRx software before screening. The active sites of both drug targets were predicted using Cavity Plus tool^52^. The predicted pocket which had native ligand binding residues was considered for grid generation in PyRx. Initially, structure based virtual Screening (SBVS) for the collected phytochemicals from each selected plant was performed using PyRx Software^53^, as reported earlier^54^. After completion of six separate SBVS experiments, viz. M^pro^-*E. angustifolia*, M^pro^-*E. purpurea*, M^pro^-*H. perforatum*, RdRp- *E. angustifolia*, RdRp-*E. purpurea*, and RdRp-*H. perforatum*, the top potential compounds with highest docking scores from SBVS were further selected for re-docking analysis in Chimera-AutoDock Vina plugin setup^55,56^. Selected ligands poses and target proteins were prepared using Dockprep tool in-built in Chimera program before re-docking. In the case of M^pro^, the grid (30 Å × 30 Å × 30 Å) was set up along the three center coordinates (119.44 × − 4.95 × 16.78), covering the entire crucial residues of the target protein to provide enough space for the ligand binding. In case of RdRp, the grid (30 Å × 30 Å × 40 Å) was set up along the three center coordinates (97.36 × 97.76 × 116.69). Similar methodology was used for docking both targets with control ligands [X-77 (M^pro^), Remdesivir (RdRp)]. Later, potential top two compounds from re-docking analysis from each plant against selected viral proteins were studied for molecular interaction analysis in free academic Schrödinger-Maestro v12.7 (Schrödinger Re-lease 2021-1: Maestro, Schrödinger, LLC, New York, NY, 2021).

#### Molecular dynamics simulation

Selected re-docked complexes from each docking group were studied under 100 ns molecular dynamics simulation to understand the binding stability of phytochemicals in the active pocket of the selected viral proteins, i.e., SARS-CoV-2 M^pro^ and SARS-CoV-2 RdRp, as reported earlier^57^. Briefly, the selected pose of docked complexes was pre-processed under default parameters using protein preparation tool in free academic Maestro-Desmond suite (Maestro-Desmond Interoperability Tools, Schrödinger, New York, NY, 2018)^58^. After that, the complexes were merged in orthorhombic water bath (10 × 10 × 10 Å) amended with TIP4P water solvent and the complete simulation system was neutralized by counter ions while ions were placed at a distance of 20 Å from the ligand using system building tool. Moreover, to mimick the in vitro physiochemical environment, 0.15 M salt was also amended in the simulation system by system building tool. Following, complete system was minimized using Desmond minimization tool^58^ under default parameters and then subjected to 100 ns MD simulation at 300 K temperature and 1.01325 bar pressure using molecular dynamics simulation tool with default parameters. Each docked complex was subjected to MD simulation under similar parameters. Subsequently, the MD simulation trajectories were analyzed by simulation interaction diagram^59^ tool in Maestro-Desmond suite^58^.

## Results

### Evaluation of cytotoxicity of *Hypericum perforatum* and *Echinacea*

The cytotoxicity of *H. perforatum*, *Echinacea* and the *H. perforatum-Echinacea* mixture were evaluated on Vero E6 cell 48 h post-treatment using MTT assay. The results showed that CC_50_ of *H. perforatum* and *H. perforatum-Echinacea* mixture were found to be: 66.78 and 141.1 µg/mL; respectively, while *Echinacea* showed the highest cytotoxicity with concentration-dependent cytotoxicity (Fig. S1).

Further evaluation of the cytotoxicity of the plant extracts was performed on HEK293 cells as a human cell line. The cytotoxicity of the extracts was not concentration-dependent with the highest cytotoxicity for the *Echinacea* extract (Fig. S1).

### Anti-viral efficacy of *Hypericum perforatum* and *Echinacea*

**Figure 2:**
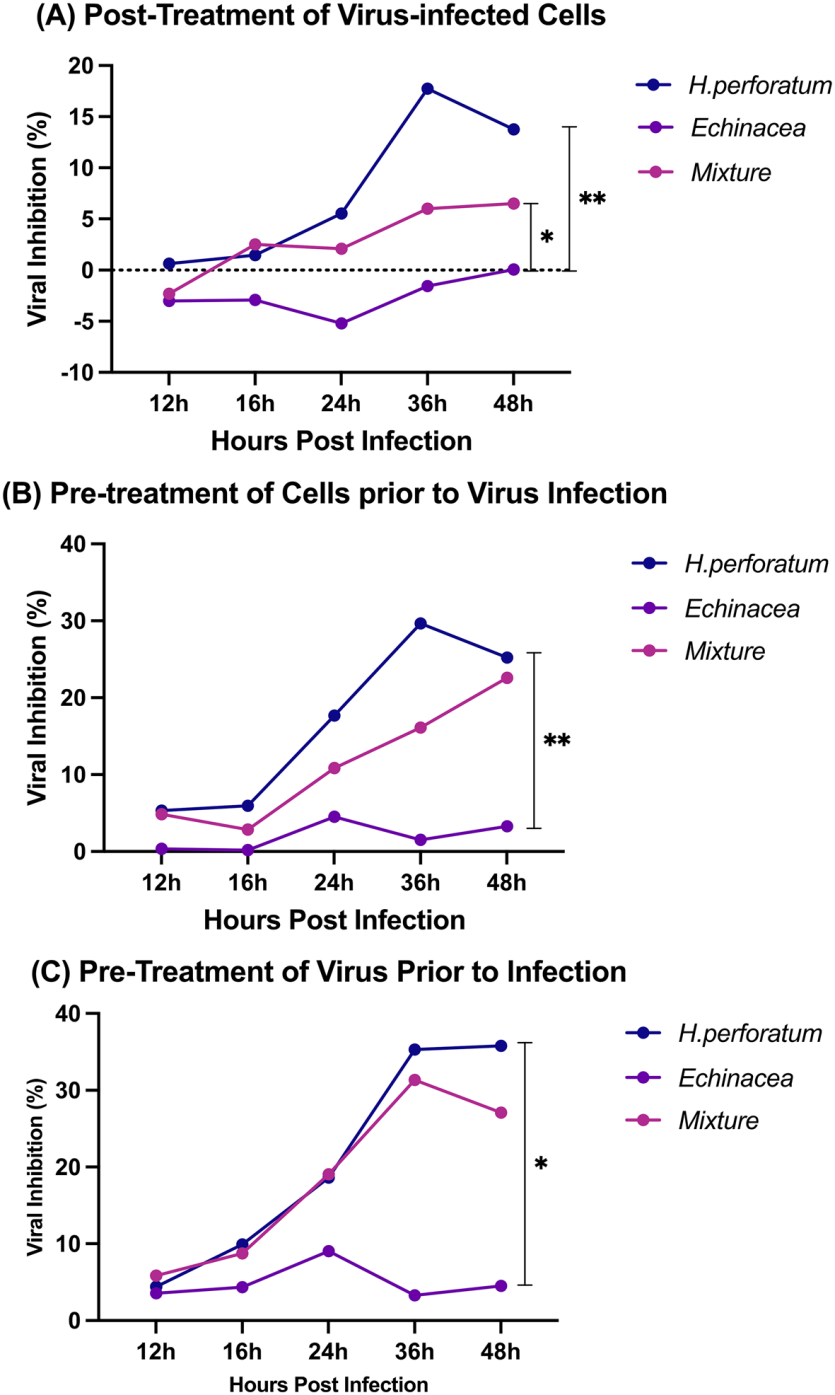
The inhibitory effect of *H. perforatum*, *Echinacea*, and the *H. perforatum-Echinacea* mixture on RNA levels of SARS-CoV-2 in Vero E6 cells was evaluated by qRT-PCR. The three experimental designs as: Post Treatment of Virus-infected Cells (**A**), Pre-treatment of Cells Prior to Virus Infection (**B**), and Virucidal (**C**) assays. The viral inhibition percentage of *H. perforatum* was 18, 30, and 36% up to 36 h in (**A**–**C**) respectively, while it was 7, 24, and 32% for the *H. perforatum-Echinacea* mixture, and it showed the least inhibition percentage with *Echinacea* up to 24 h. The inhibitory effect was representatives of two independent experiments performed in triplicate. Statistical analysis showed that differences were significant with *p* < 0.05 and *p* < 0.005 (one- & two-way ANOVA).

**Figure 3:**
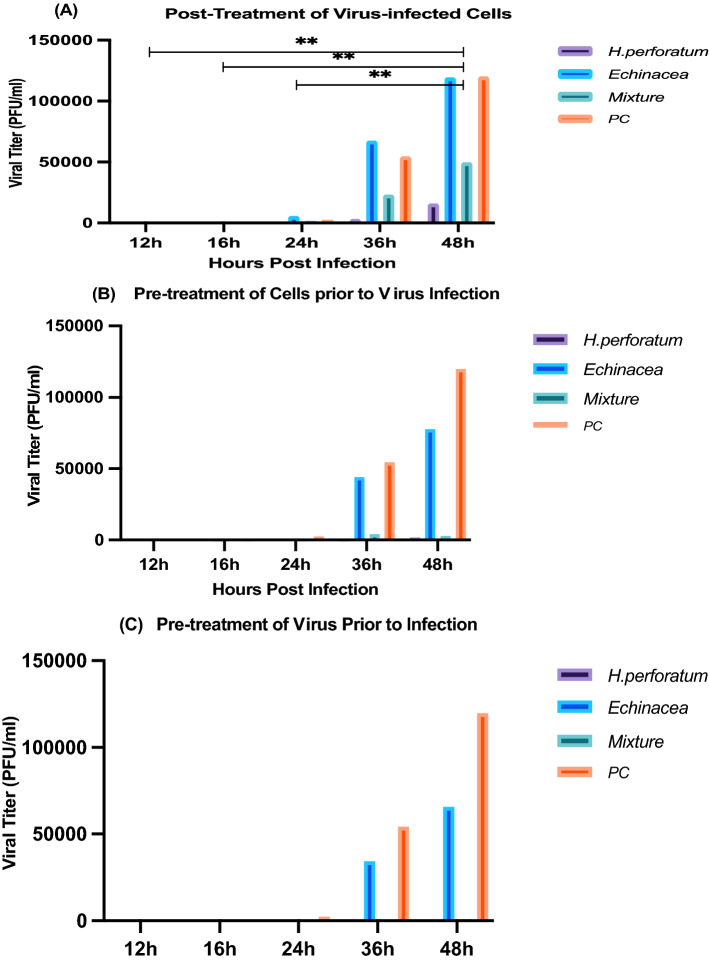
The effect of *H. perforatum*, *Echinacea*, and *H. perforatum-Echinacea* mixture on viral load of SARS-CoV-2 infection in Vero E6 cells was evaluated by qRT-PCR. The three experimental designs: post treatment of virus-infected cells (**A**), pre-treatment of cells prior to virus infection (**B**), and virucidal (**C**) assays. Comparing to the viral control, the viral load of *H. perforatum* was less than 25,000 PFU/mL up to 48 h in (**A**) and almost zero in (**B**) and (**C**), while it was less than 50,000 PFU/mL for the H. *perforatum-Echinacea* mixture, and it showed the most viral load with *Echinacea* up to 36 h. The viral load was representatives of two independent experiments performed in triplicate. Statistical analysis showed that differences were significant with *p* < 0.005 (one- & two-way ANOVA), PC is a positive virus control where no treatment is applied.

The antiviral effect of the medicinal plants was evaluated using qRT-PCR assay relative to the virus control as shown in Fig. S2 which shows the dose–response curve for the tested plants. The time response of the plants tested were evaluated through the following assays:

1. *Post-treatment of virus-infected cells assay* Results from this assay showed that *H. perforatum* had the highest efficacy (Fig. 2A) with a minimum inhibitory concentration (MIC) of 1.56 µg/mL while the *H. perforatum-Echinacea* mixture showed an MIC of 6.25 µg/mL and the least effective was *Echinacea* of 6.25 µg/mL. Following the viral load with time (12, 18, 24, 36, 48 h) (Fig. 3A) after the addition of the mixture showed a significant reduction of the viral load for *H. perforatum* up to 36 h of addition (*p* = 0.0047) followed by the *H. perforatum-Echinacea* mixture up to 36 h of addition (*p* = 0.0048) and *Echinacea* up to 24 h of addition (*p* = 0.0060).
2. Pre-treatment of cells prior to viral infection assay: The outcome of this assay showed that *H. perforatum* had an MIC of 1.56 µg/mL, while *Echinacea* and the *H. perforatum-Echinacea* mixture had an MIC of 6.25 µg/mL. Figures 2B and 3B (represented previously) showed the efficacy in the viral inhibition (%) and the reduction in the viral load, respectively, with *H. perforatum*, the *H. perforatum-Echinacea* mixture and *Echinacea* over time (12–48 h).
3. Virucidal activity assay (Pre-treatment of virus prior to infection): Results from this assay showed that *H. perforatum* had the highest effect (Figs. 2C and 5c) of 1.56 µg/mL followed by the *H. perforatum-Echinacea* mixture then *Echinacea* of 6.25 µg/mL. The time of addition of the drug (during the infection cycle) showed a significant reduction in the viral load for *H. perforatum* that lasted longer than 48 h, and this result was shown as well with the *H. perforatum-Echinacea* mixture; however, *Echinacea* displayed a virucidal effect only up to 36 h.

**Figure 4:**
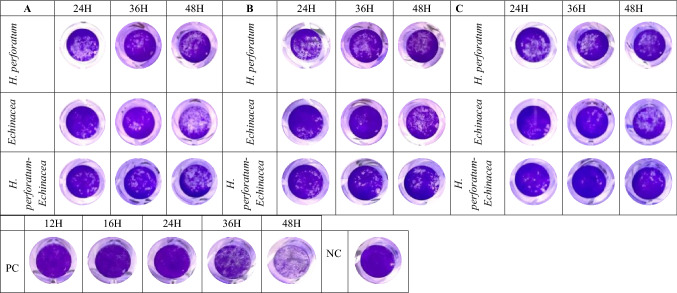
The impact of *H. perforatum*, *Echinacea* and the *H. perforatum-Echinacea* mixture on SARS-CoV-2 infected-Vero E6 cells by anti-viral activity essays (**A**,**B**,**C**). The cell viability and CPE through SARS-CoV-2 infectious cycle, from 24 to 48 h post infection, were visualized by photographed images by the crystal violet assay (blue circles). The images were captured when the CPE started on the positive control at 24 h. The results were representatives of two independent experiments and performed in triplicate.

The anti-viral effect of *H. perforatum*, *Echinacea* and the *H. perforatum-Echinacea* mixture (Figs. 2 and 3) was evaluated in maximum non-toxic concentration (1.56, 6.25, and 6.25 µg/mL, respectively), and it showed that the inhibition of *H. perforatum* on SARS-CoV-2 was higher than *H. perforatum-Echinacea* mixture and *Echinacea* in the three anti-viral assays. Also, it displayed that *Echinacea* was a weaker inhibitor than the *H. perforatum* and the *H. perforatum-Echinacea* mixture but slightly strong as a virucidal effect up to 24 h. In addition, the effect of the plant materials on SARS-CoV-2 infection was evaluated by crystal violet staining of the viral CPE effect on the cells (Fig. 4). Figure 4 shows the effect of evaluating the antiviral activity of adding the extracts in the three different antiviral assays (Panels A, B and C) and followed up for 48 h post addition. PC is the positive virus control with no treatments added, while NC is the negative cell control with no virus and no treatment added.

### Computational analysis

To decipher the potential compounds, present in the respective extracts of selected plants, phytochemicals reported in each plant were collected from literature and studied for putative inhibitory mechanism with the aid of computational methods. This aid includes molecular docking, molecular contact formation, and molecular dynamics simulation.

#### Data collection, virtual screening, and re-docking

SBVS of total 394 phytochemicals from *E. angustifolia*, *E. purpurea*, and *H. perforatum* against SARS-CoV-2 M^pro^ and SARS-CoV-2 RdRp predicted their binding affinity between − 11 and − 1 kcal/mol (Tables S1–S6). Following, top 10 compounds from each SBVS of M^pro^-*E. angustifolia*, M^pro^-*E. purpurea*, M^pro^-*H. perforatum*, RdRp-*E. angustifolia*, RdRp-*E. purpurea*, and RdRp-*H. perforatum* were selected for re-docking analysis. The binding energies of re-docked molecules from each plant against both drug targets have been listed in Tables S7–S12. In the case of SARS-CoV-2 M^pro^ re-docking analysis, *Echinacin* (− 9.0 kcal/mol) and Echinacoside (− 8.6 kcal/mol) from *E. angustifolia*, Cyanin (− 9.6 kcal/mol) and Cyanidin 3-(6''-malonylglucoside) (− 9.0 kcal/mol) from *E. purpurea*, and Querce-tin-3-O-glucuronide (− 9.6 kcal/mol) and Proanthocyanidins (− 9.1 kcal/mol) from *H. perforatum* showed substantial binding energy with viral protease and much better than the native ligand X77 (− 8.4 kcal/mol).

While, in the case of SARS-CoV-2 RdRp re-docking analysis Echinacoside (− 9.0 kcal/mol) and Rutin (− 8.0 kcal/mol) from *E. angustifolia*, Kaempferol-3-O-rutinoside (− 9.3 kcal/mol) and Echinacoside (− 9.2 kcal/mol) from *E. purpurea*, and Rutin (− 9.2 kcal/mol) and Quercetin-3-O-xyloside (− 9.0 kcal/mol) from *H. perforatum* showed significant binding energy with the target protein. These observed binding energies for the above compounds are much better than control ligand Remdesivir (− 7.6 kcal/mol), and some previously reported FDA approved drugs, which were repurposed against SARS-CoV-2^60,61^. This suggests that all the above reported compounds may be the potential inhibitors of both SARS-CoV-2 drug targets. The molecular interaction analysis results revealed the formation of good molecular contacts with catalytic residues and other substrate binding residues (Tables 1 and 2).

**Table 1:** Molecular interaction analysis of SARS-CoV-2 M^pro^ with screened phytochemicals from selected plants.

S. no.	Compound, PubChem ID, & source	H-bond	π–π stacking	Hydrophobic	Polar	Negative	Positive	Glycine
1	Echinacin (6439941) (*E. angustifolia*)	Phe^140^, His^163^, Glu^166^, Gln^192^	–	Pro^168^, Leu^167^, Met^165^, Phe^140^, Leu^141^, Cys^145^, Met^49^, Cys^44^, Pro^52^, Tyr^54^	His^172^, Asn^142^, Ser^144^, Thr^26^, Thr^25^, His^41^, Gln^189^, Thr^190^, Gln^192^, His^164^, His^163^	Glu^166^, Asp^187^	Arg^188^	Gly^143^
2	Echinacoside (5281771) (*E. angustifolia*)	Thr^190^, Arg^188^, Asn^142^, Glu^166^, Gly^143^, Thr^26^, Thr^25^, His^41^, Cys^44^	–	Ala^191^, Phe^140^, Leu^141^, Pro^168^, Met^165^, Cys^145^, Cys^44^, Val^42^, Met^49^	Gln^192^, Thr^190^, Gln^189^, Hie^172^, Asn^142^, Ser^144^, Hie^164^, His^163^, Asn^119^, Thr^26^, Thr^25^, Thr^24^, His^41^, Thr^45^, Ser^46^	Glu^166^	Arg^188^	Gly^143^
3	Cyanin (441688) (*E. purpurea*)	Tyr^54^, Thr^190^, Cys^44^, Glu^166^, Hie^163^, Asn^142^	–	Ala^191^, Pro^168^, Leu^167^, Met^165^, Phe^140^, Leu^141^, Cys^145^, Cys^44^, Met^49^, Pro^52^, Tyr^54^	Gln^189^, Thr^190^, Gln^192^, Hie^164^, Hie^163^, Asn^142^, Hie^172^, Ser^144^, Hie^41^, Thr^45^, Ser^46^, Thr^25^	Glu^166^, Asp^187^	Arg^188^	Gly^143^
4	Cyanidin 3-(6''-alonylglucoside (44256740) (*E. purpurea*)	Glu^166^, Gln^189^, Asp^187^, Cys^44^	–	Phe^140^, Leu^141^, Cys^145^, Met^165^, Leu^167^, Pro^168^, Ala^191^, Cys^44^, Tyr^54^, Pro^52^, Met^49^	Asn^142^, Ser^144^, Hie^163^, Gln^192^, Thr^190^, Gln^189^, Hie^41^	Glu^166^, Asp^187^	Arg^188^	Gly^143^
5	Quercetin-3-O-glucuronide (5274585) (*H. perforatum*)	His^163^, Asn^142^, Glu^166^, Met^49^, Cys^44^, Thr^190^	–	Pro^168^, Leu^167^, Met^165^, Leu^141^, Cys^145^, Tyr^54^, Pro^52^, Cys^44^, Met^49^, Ala^191^	His^163^, Asn^142^, His^172^, Ser^144^, His^41^, Gln^189^, Thr^190^ Gln^192^	Glu^166^, Asp^187^	Arg^188^	Gly^143^
6	Proanthocyanidins (107876) (*H. perforatum*)	Arg^188^, Thr^190^, Glu^166^, Asn^142^, Ser^144^	His^41^	Cys^44^, Met^49^, Tyr^54^, Ala^191^, Pro^168^, Met^165^, Phe^140^, Leu^141^, Cys^145^	His^41^, Gln^189^, Thr^190^, Gln^192^, His^164^, His^163^, Asn^142^, Ser^144^	Glu^166^, Asp^187^	Arg^188^	–
7	M^pro^-X77 (Control)	Gly^143^, His^163^, Glu^166^	His^41^	Leu^27^, Cys^44^, Met^49^, Pro^52^, Tyr^54^, Phe^140^, Leu^141^, Cys^145^, Met^165^, Leu^167^, Pro^168^	Thr^25^, Thr^26^, Asn^142^, Ser^144^, His^163^, His^164^, His^172^, Gln^189^, Thr^190^, Gln^192^	Glu^166^, Asp^187^	His^41^	Gly^143^

**Table 2:** Molecular interaction analysis of SARS-CoV-2 RDRP with selected phytochemicals from selected plants.

S. no.	Compound, PubChem ID, & source	H-bond	π–π/*π–π cation	Hydrophobic	Polar	Negative	Positive	Glycine
1	Echinacoside (5281771) (*E.* *angustifolia)*	Asp^452^, Thr^687^, Asp^760^, Asp^618^, Lys^551^, Arg^553^, Ala^554^, Asp^623^	–	Ala^688^, Tyr^455^, Cys^622^, Ala^554^, Pro^620^, Tyr^619^, Met^794^, Phe^793^, Val^166^	Ser^814^, Ser^795^, Thr^556^, Ser^682^, Thr^687^, Asn^691^, Ser^759^	Asp^452^, Asp^623^, Asp^618^, Asp^164^, Glu^167^, Asp^761^, Asp^760^	Arg^624^, Arg^555^, Lys^621^, Arg^553^, Lys^551^, Lys^798^	–
2	Rutin (5280805) (*E. angustifolia)*	Asp^760^, Asp^452^, Arg^555^, Arg^553^, Lys^551^, Asp^618^	*Arg^555^, *Lys^551^	Cys^622^, Pro^620^, Tyr^619^, Ala^550^, Ala^554^	Ser^549^, Thr^556^	Asp^760^, Asp^623^, Asp^618^, Asp^452^	Arg^624^, Lys^621^, Lys^798^, Lys^551^, Arg^553^, Arg^555^	–
3	Kaempferol-3-O-rutinoside (5318767) (*E. purpurea*)	Asp^452^, Lys^551^, Asp^623^, Lys^621^, Tyr^619^, Asp^618^, Asp^760^,	–	Ala^554^, Ala^550^, Cys^622^, Pro^620^, Tyr^619^, Trp^617^, Ala^762^, Cys^813^, Tyr^455^	Ser^814^, Ser^549^, Thr^556^	Asp^452^, Glu^811^, Asp^761^, Asp^760^, Asp^618^, Asp^623^	Arg^555^, Arg^553^, Lys^551^, Arg^624^, Lys^621^, Lys^798^	
4	Echinacoside (5281771) (*E. purpurea*)	Ser^795^, Asp^164^, Asp^760^, Thr^687^, Asp^623^, Asp^452^, Arg^555^, Ala^554^, Arg^553^	–	Val^166^, Phe^793^, Ala^554^, Tyr^619^, Pro^620^, Tyr^455^, Cys^622^, Ala^688^	Ser^795^, Ser^814^, Ser^759^, Asn^691^, Ser^682^, Thr^687^, Thr^556^	Asp^618^, Asp^164^, Asp^623^, Asp^452^, Asp^761^, Asp^760^	Lys^798^, Lys^551^, Arg^553^, Arg^555^, Lys^621^, Arg^624^	–
5	Rutin (5280805) *(H. perforatum)*	Asp^760^, Asp^452^, Arg^555^, Arg^553^, Lys^551^, Asp^618^	*Arg^555^, *Lys^551^	Cys^622^, Pro^620^, Tyr^619^, Ala^550^, Ala^554^	Ser^549^, Thr^556^	Asp^760^, Asp^623^, Asp^618^, Asp^452^	Arg^624^, Lys^621^, Lys^798^, Lys^551^, Arg^553^, Arg^555^	–
6	Quercetin-3-O-xyloside (5320863) *(H. perforatum)*	Asn^497^, Arg^569^, Gly^683^, Asn^543^	*Lys^500^	Ala^502^, Val^557^, Ala^558^, Val^560^, Ile^562^, Leu^498^, Val^495^, Ala^512^, Tyr^516^, Ala^685^	Asn^543^, Ser^501^, Asn^497^, Thr^565^	Asp^684^	Arg^569^, Lys^500^	Gly^683^, Gly^559^
7	M^pro^-remdesivir (control)	Arg^553^, Thr^556^, Cys^622^, Asp^623^, Asn^691^,Ser^759^	–	Tyr^455^, Ala^554^, Val^557^, Tyr^619^, Pro^620^, Cys^622^, Ala^688^	Ser^549^, Thr^556^, Thr^680^, Ser^682^, Thr^687^, Asn^691^, Ser^759^	Asp^618^, Asp^623^, Asp^760^	Lys^545^, Lys^551^ Arg^553^, Arg^555^, Lys^621^, Arg^624^	–

**Figure 5:**
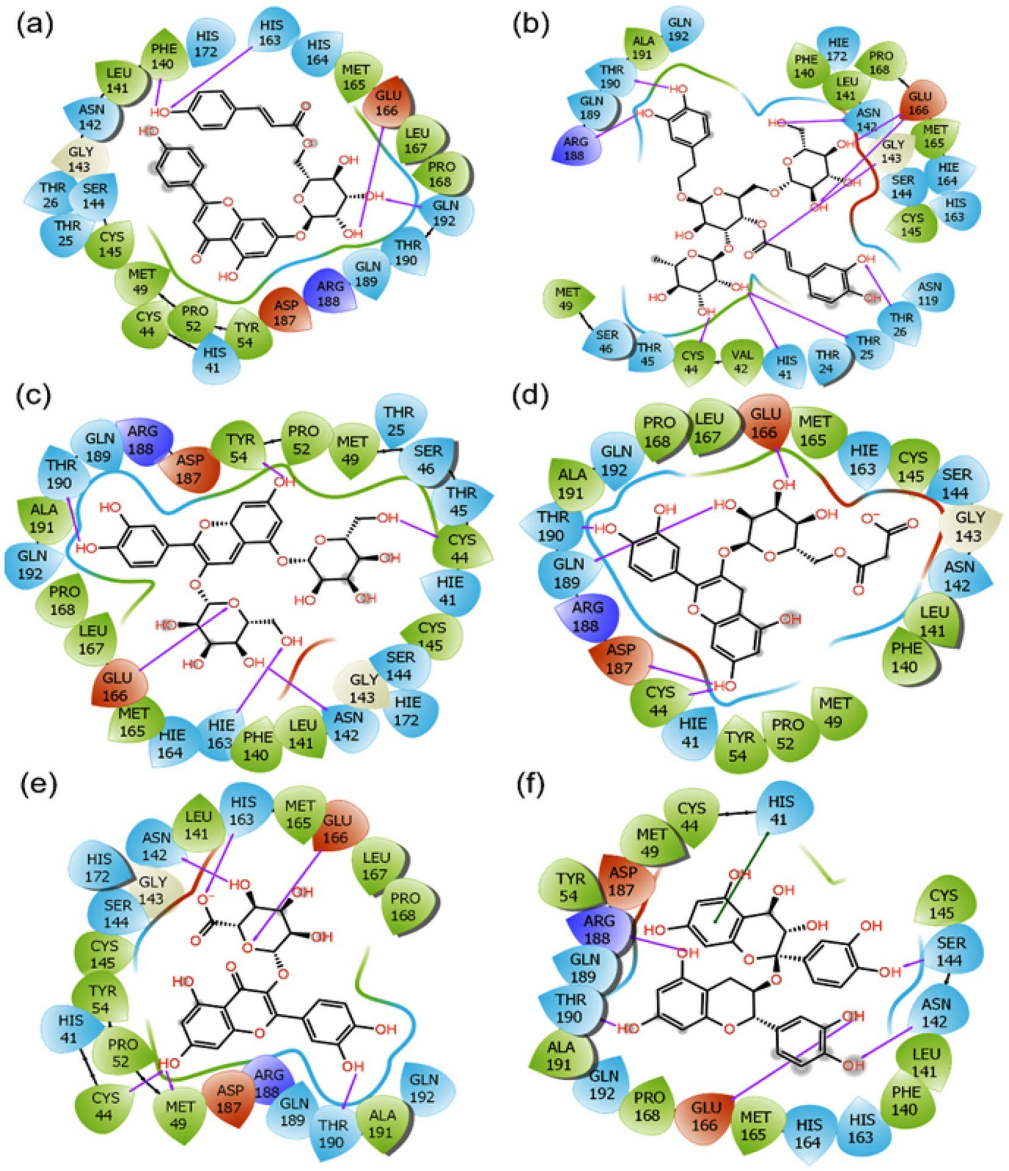
2D molecular contact profiling for the re-docked poses of SARS-CoV-2 M^pro^ with selected phytochemicals from selected plants, viz. (**a**) SARS-CoV-2 M^pro^-Echinacin, (**b**) SARS-CoV-2 M^pro^-Echinacoside, (**c**) SARS-CoV-2 M^pro^-Cyanin, (**d**) SARS-CoV-2 M^pro^-Cyanidin 3-(6ʺ-alonylglucoside), (**e**) SARS-CoV-2 Mpro-Quercetin-3-O-glucuronide, and (**f**) SARS-CoV-2 M^pro^-Proanthocyanidins. These poses exhibited hydrogen bond (pink arrows), π–π (green lines), hydrophobic^55^, polar (blue), negative (red), positive (violet), and glycine (grey) intermolecular interactions for the respective docked complexes.

Intermolecular interactions were also assessed for the respective docked complexes of SARS-CoV-2 M^pro^ (Table 1). Notably, SARS-CoV-2 M^pro^-Echinacin complex analysis, four hydrogen bonds formation were observed with Echinacin and Phe^140^, His^163^, Glu^166^, and Gln^192^ residues of SARS-CoV-2 M^pro^ while Thr^190^, Arg^188^, Asn^142^, Glu^166^, Gly^143^, Thr^26^, Thr^25^, His^41^, and Cys^44^ residues of SARS-CoV-2 M^pro^ formed ten hydrogen bonds with Echinacoside from *E. angustifolia* (Fig. 5). Cyanin from *E. purpurea* formed six hydrogen bonds with Tyr^54^, Thr^190^, Cys^44^, Glu^166^, Hie^163^, and Asn^142^ while Cyanidin 3-(6ʺ-malonylglucoside) formed five bonds with Glu^166^, Gln^189^, Asp^187^, and Cys^44^ residues of SARS-CoV-2 M^pro^ (Fig. 5). Quercetin-3-O-glucuronide from *H. perforatum* formed six hydrogen bonds with His^163^, Asn^142^, Glu^166^, Met^49^, Cys^44^, and Thr^190^ while Proanthocyanidins formed five hydrogen bonds with Arg^188^, Thr^190^, Glu^166^, Asn^142^, and Ser^144^ residues of SARS-CoV-2 M^pro^ (Fig. 5). The observed interaction for each above discussed molecule is much better than the interaction reported in the crystal structure of SARS-CoV-2 M^pro^ bound to potent broad-spectrum non-covalent inhibitor X77 because only three hydrogen bonds (with Gly^143^, His^163^, and Glu^166^) had been observed in this determined structure of the complex and also in re-docked complex of SARS-CoV-2 M^pro^ -X77 (Fig. S5)^50^.

**Figure 6:**
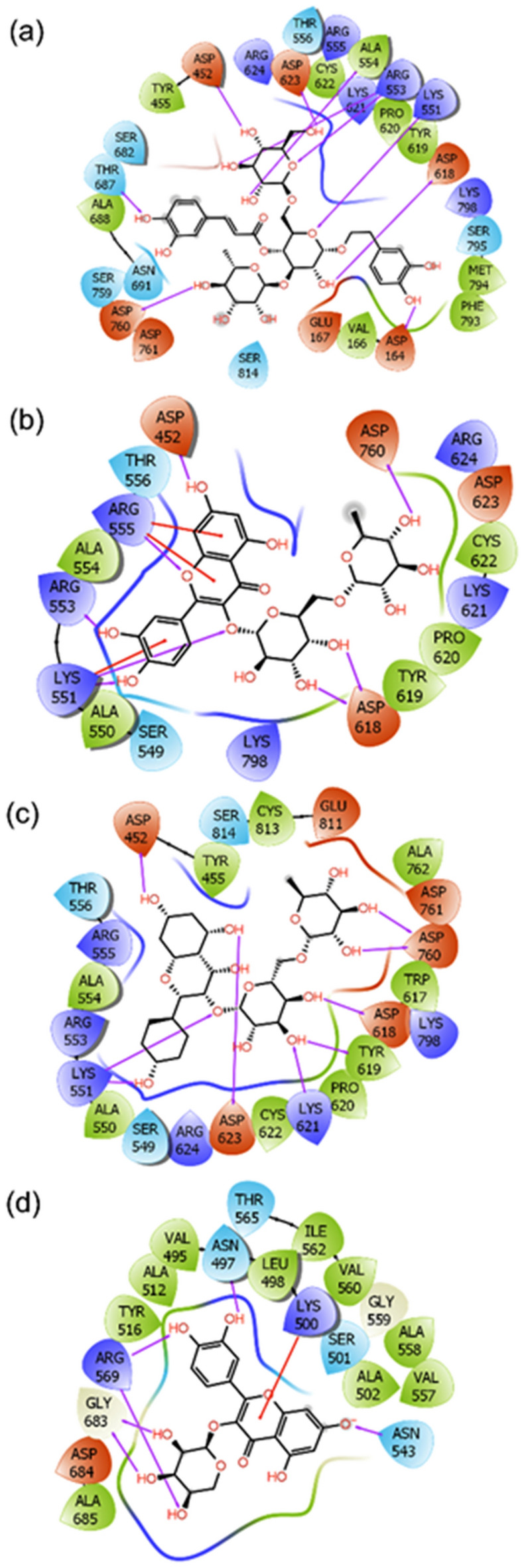
2D molecular contact profiling for the re-docked poses of SARS-CoV-2 Rdrp with selected phytochemicals from selected plants, viz. (**a**) SARS-CoV-2 RdRp-Echinacoside, (**b**) SARS-CoV-2 RdRp-Rutin, (**c**) SARS-C0V-2 Rdrp-Kaempferol-3-O-rutinoside, and (**d**) SARS-CoV-2 Rdrp-Quercetin-3-o-xyloside. These poses exhibited hydrogen bond (pink arrows), π–π (green lines), hydrophobic^55^, polar (blue), negative (red), positive (violet), and glycine (grey) intermolecular interactions for the respective docked complexes.

Likewise, interactions for the selected docked poses of SARS-CoV-2 RdRp with selected phytochemicals were also studied (Table 2). However, Interestingly, SARS-CoV-2 RdRp-Echinacoside showed 10 hydrogen bonds formation at Asp^452^, Thr^687^, Asp^760^, Asp^618^, Lys^551^, Arg^553^, Ala^554^, and Asp^623^ residues while Asp^760^, Asp^452^, Arg^555^, Arg^553^, Lys^551^, and Asp^618^ residues of SARS-CoV-2 RdRp formed 8 hydrogen bonds and three π-cation contacts (Arg^555^, and Lys^551^) with Rutin from *E. angustifolia* (Fig. 6). In this study, Kaempferol-3-O-rutinoside from *E. purpurea* formed nine hydrogen bonds with Asp^452^, Lys^551^, Asp^623^, Lys^621^, Tyr^619^, Asp^618^, and Asp^760^ residues of SARS-CoV-2 RdRp while Quercetin-3-O-xyloside *from H. perforatum* formed six hydrogen bonds and (with Asn^497^, Arg^569^, Gly^683^, Asn^543^) and one pi-cation interaction (Lys^500^) with SARS-CoV-2 RdRp (Fig. 6). Notably, a significant role of hydrogen bond formation between the receptor and ligand in the complex stability has been discussed and established in the field of drug discovery (please cite, Patil R, Das S, Stanley A, Yadav L, Sudhakar A, Varma AK. Optimized hydrophobic interactions and hydrogen bonding at the target-ligand interface leads the pathways of drug-designing^62,63^. Altogether, the calculated docking scores and intermolecular contact formation between RdRp and respective phytochemicals from *E. angustifolia*, *E. purpurea*, and *H. perforatum* suggested the screened compounds as potential inhibitors of SARS-CoV-2 RdRp by comparison to Remdesivir, potential inhibitors of SARS-CoV-2 RdRp, which was also reported with − 7.6 kcal/mol docking scores and interactions with similar active residues in SARS-CoV-2 RdRp^58,60,61^.

#### Molecular dynamics simulation analysis

In the area of drug discovery, molecular dynamics simulation of the screened complexes from molecular docking is performed to understand the respective complex stability and intermolecular interactions with respect to time. In this study, selected docked complexes were studied for MD simulation interval via root mean square deviation (RMSD), root mean square fluctuation (RMSF), and protein–ligand contacts mapping as function of 100 ns simulation interval.

**Figure 7:**
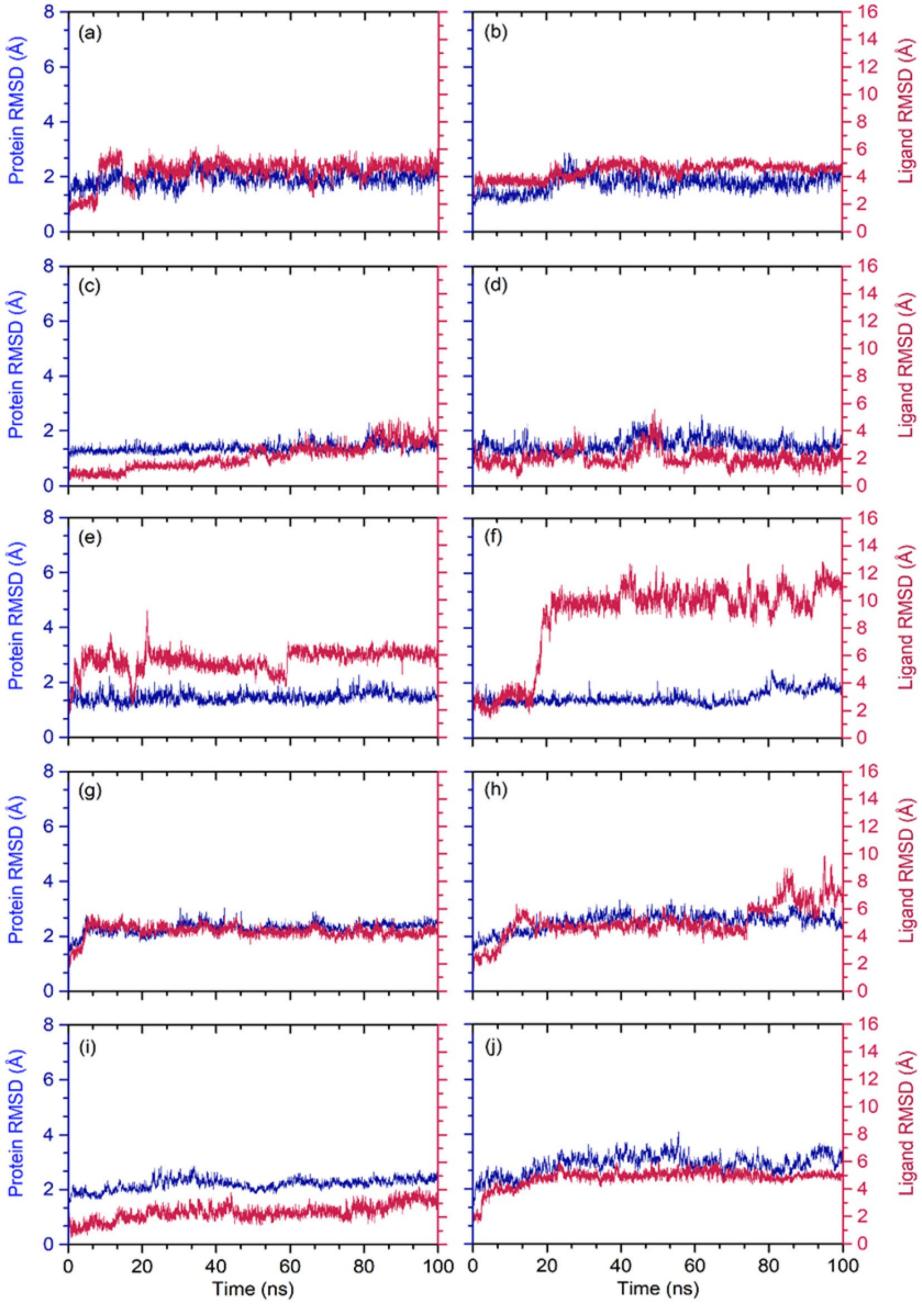
Calculated RMSD values for alpha carbon (Cα) atoms (blue curves) of SARS-CoV-2 proteins and docked ligands (red curves), viz. (**a**) SARS-CoV-2 M^pro^-Echinacin, (**b**) SARS-CoV-2 M^pro^-Echinacoside, (**c**) SARS-CoV-2 M^pro^-Cyanin, (**d**) SARS-CoV-2 M^pro^-Cyanidin 3-(6ʺ-alonylglucoside), (**e**) SARS-CoV-2 Mpro-Quercetin-3-O-glucuronide, and (**f**) SARS-CoV-2 M^pro^- Proanthocyanidins, (**g**) SARS-CoV-2 RdRp-Echinacoside, (**h**) SARS-CoV-2 RdRp-Rutin, (**i**) SARS-CoV-2 RdRp-Kaempferol-3-O-rutinoside, and (**j**) SARS-CoV-2 RdRp-Quercetin-3-O-xyloside, were plotted with respect to 100 ns simulation interval.

RMSD analysis from MD trajectory of docked complex can be useful to understand the convergence of the complex with respect to time. Initially, protein (Cα) and protein fit ligands, i.e. selected phytochemical from three plants, were collected from the respective MD trajectories (Fig. 7). Notably, calculated RMSD values for alpha carbon (Cα) atoms of both SARS-CoV-2 M^pro^ and SARS-CoV-2 RdRp showed < 3 Å acceptable deviations throughout the course of simulation. These observations suggested that the docked viral proteins have no significant structural changes as function of 100 ns interval (Fig. 7). Likewise, all the selected phytochemical as protein fit ligands with respective viral proteins showed substantial stability of < 5 Å, except SARS-CoV-2 M^pro^-Quercetin-3-O-glucuronide (< 6.5 Å), SARS-CoV-2 M^pro^-Proanthocyanidins (< 11.3 Å), and SARS-CoV-2 RdRp-Rutin (< 7.5 Å) exhibited higher deviations at the end of 100 ns simulation interval, suggested the substantial stability of the docked complexes (Fig. 7). Furthermore, these observations were also supported by acceptable fluctuations in protein RMSF (< 3 Å for SARS-CoV-2 M^pro^ and < 6 Å SARS-CoV-2 RdRp) and protein fit ligand RMSF (< 3 Å in both proteins) values for the respective docked complexes, except higher < 6 Å deviations in SARS-CoV-2 RdRp in the region 300–325 residues in SARS-CoV-2 RdRp-Echinacoside and C-terminal in of the viral RdRp (Figs. S3–S4). Collectively, RMSD and RMSF analysis of the docked complexes supports the stability of the docked complexes in the active pocket of viral protease and RdRp during 100 ns MD simulation.

**Figure 8:**
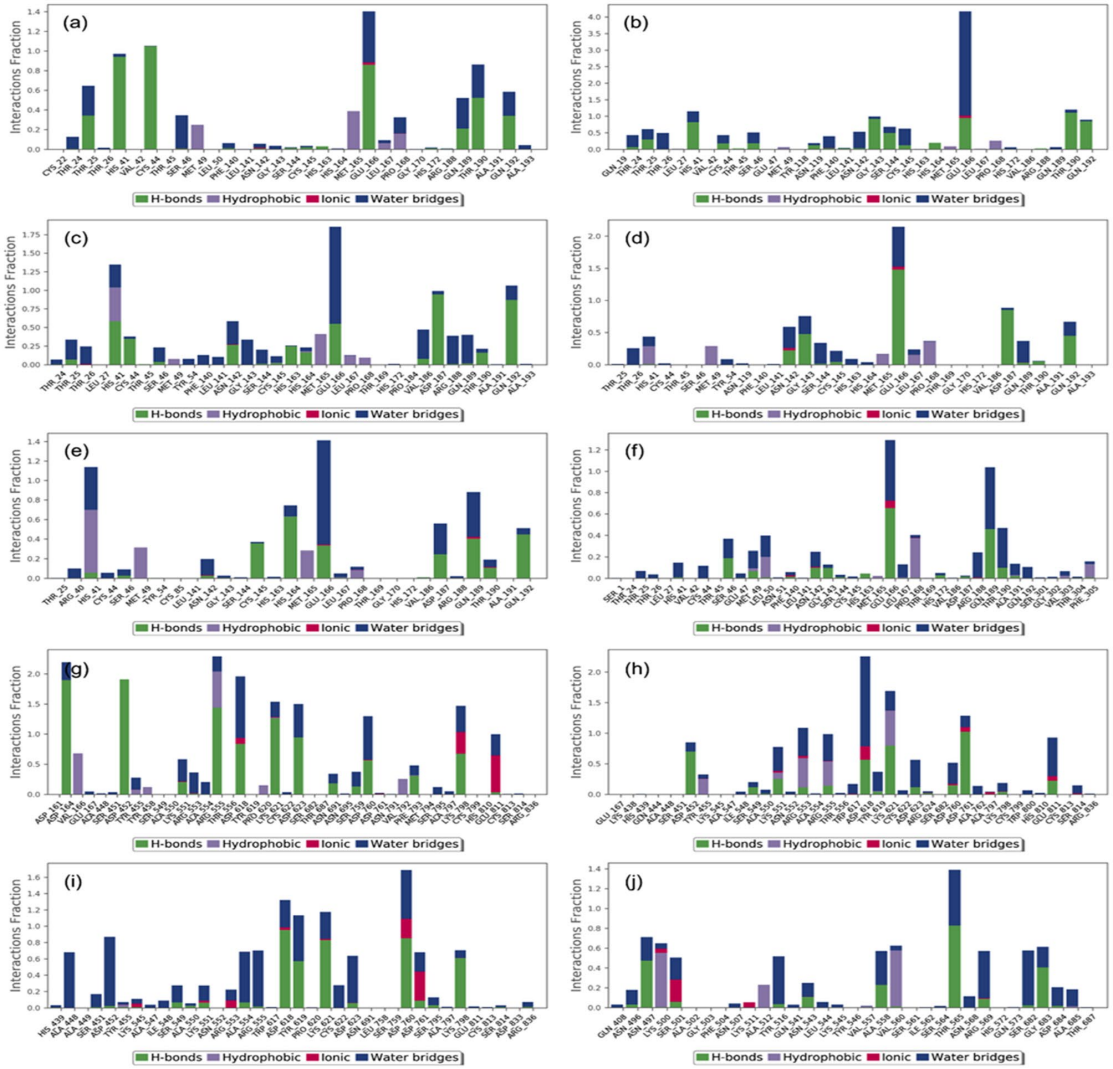
Protein–ligand interactions mapping for SARS-CoV-2 proteins with docked ligands, i.e., (**a**) SARS-CoV-2 M^pro^-Echinacin, (**b**) SARS-CoV-2 M^pro^-Echinacoside, (**c**) SARS-CoV-2 M^pro^-Cyanin, (**d**) SARS-CoV-2 M^pro^-Cyanidin 3-(6ʺ-alonylglucoside), (**e**) SARS-CoV-2 M^pro^-Quercetin-3-O-glucuronide, and (**f**) SARS-CoV-2 M^pro^- Proanthocyanidins, (**g**) SARS-CoV-2 RdRp-Echinacoside, (**h**) SARS-CoV-2 RdRp-Rutin, (**i**) SARS-CoV-2 RdRp-Kaempferol-3-O-rutinoside, and (**j**) SARS-CoV-2 RdRp-Quercetin-3-O-xyloside, extracted from respective 100 ns molecular dynamics simulations trajectories.

To further assess the stability of the docked complexes, protein-ligand interaction profiles, which include hydrogen bonding, hydrophobic interactions, ionic interactions, and water bridge formation, were extracted from the respective MD simulation trajectories (Fig. 8). Notably, the simulated complexes exhibited considerable molecular contact formation with the essential residues in the active pockets of the viral proteins during 100 ns simulation interval. Interestingly, the interacting residues were also noted in the initial docked poses of respective complexes (Tables 1,2). Of note, A significant contribution of hydrogen bond formation and water bridge assimilations were noted in SARS-CoV-2 M^pro^ with selected phytochemicals while a substantial contribution of ionic interactions were also noted be-sides hydrogen and water bridge formation in SARS-CoV-2 RdRp docked with selected phytochemicals during simulation interval (Fig. 8). These protein-ligand contact maps, hence, suggested the considerable stability of docked ligands by formation of intermolecular contacts with the active residues of the viral proteins. Therefore, analysis of MD simulation suggested the selected phytochemicals, i.e. Echinacin, Echinacoside, Cyanin, Cyanidin 3-(6ʺ-alonylglucoside), Quercetin-3-O-glucuronide, Proanthocyanidins, Echinacoside, Rutin, Kaempferol-3-O-rutinoside, and Quercetin-3-O-xyloside, as key components in the extracts of the selected plants and responsible for imposing the inhibition of the SARS-CoV-2 as observed in the in vitro experimental studies.

## Discussion

COVID-19 pandemic is causing a global challenge to the world economic, social and healthcare systems. The pandemic is responsible for the death of over 5 million confirmed cases and 288 million infections worldwide^64^. Although some vaccines are developed and are now being utilized under emergency use because of the pandemic, the efficacy of the vaccines is still debatable especially with the emergence of new variants of concern in the genomic structure^63–66^. Several non-specific treatment options were evaluated and entered clinical trials including the repurposing of known treatments against other diseases^3^. Therefore, improvement and investigation of an effective antiviral therapy is an urgent need for treating SARS-CoV-2 infection.

In our study, the anti-SARS-CoV-2 effect of the medicinal plants *H. perforatum*, *Echinacea* and their combination was evaluated. The medicinal plants were purchased from a commercial source to ensure consistency of the composition and were tested either individually or combined together as a single treatment. Their mode of action was evaluated using three approaches namely post treatment of virus–infected cells, pre-treatment of cells prior viral infection and virucidal approaches. The cytotoxic effect of the tested medicinal plants was evaluated using MTT assay with results presented as percent of cytotoxicity relative to cell control (cells with no added tested medicinal plants). The CC_50_ of *H. perforatum* and *H. perforatum-Echinacea* mixture was as follows 66.78 and 141.1 µg/mL; respectively while *Echinacea* was highly toxic.

The anti-viral activity of *H. perforatum* extract and hyperforin was previously reported to reduce the expression level of mRNA of IL-6 and TNF-α and to have a potent effect on the prevention of pro-inflammatory effect of numerous cytokines^20,23,63^. When exposed to light, hypericin showed different modes of anti-viral activities such as inhibition of budding of new virions^64^ cross-linking of capsids preventing viral uncoating^65^, and inhibition of protein kinase activity required for replication of a number of viruses^66,67^. Moreover, it binds to phospholipids such as phosphatidylcholine of cell membranes, and it binds to retroviral particles, maybe by correlating with the membrane-derived lipid envelope^68^. Gibbons et al. noted that many hypericum species include biologically active acyl phloroglucinols^69,70^. Furthermore, polycyclic quinone of hypericin were reported to have an effective light-induced antiviral activity against many of enveloped viruses, including HIV-1^64,65,71–73^. The molecular site of action of hypericin is increased > 100-fold in the presence of light^64,65,71–73^ since it is a photosensitizing compound^74^. During illumination, singlet oxygen is efficiently produced with a quantum yield of 0.73^75^ which is suggested to be the causative agent of hypericin's antiviral activity^64,65,72^ or from complex mechanisms involving the superoxide anion and hypericin^76^. Other studies found^64,65^ hypericin induces significant changes in the HIV capsid protein p24, in the presence of light, and may suppress reverse transcriptase activity.

Our results demonstrated that the *H. perforatum*, containing 0.9 mg/capsule of hypericins, can significantly reduce SARS-CoV-2 viral load compared to the positive control through the viral infectious cycle from 0 to 48 h (Table S13, Figs. 2, 3). This reduction is demonstrated in three mechanisms investigated in this study. Noticeably the results showed that the highest antiviral effect was through the virucidal mechanism (Fig. 3) compared to the other two mechanisms with the highest inhibition observed at 36 h after infection as shown in Fig. 2. While *H. perforatum* has the strongest inhibitory effect (35.77%) and reduction in viral load, up to 48 h, compared to *Echinacea* and the *H. perforatum–Echinacea* mixture that had 3.30 and 31.36%, up to 36 h, respectively. The lower inhibition of *H. perforatum–Echinacea* mixture than *H. perforatum* alone that would be expected as the active compounds in *H. perforatum* were diluted by the addition of *Echinacea.*

Hypericins were reported to be the active anti-viral compounds in *H. perforatum* extract against several viral infections both in vitro and in vivo including IBV^17^, bovine diarrhea virus (BVDV)^77^ and hepatitis C virus (HCV)^47^ with hypericin defined as the active ingredient. Other studies showed that *H. perforatum* extract had anti-viral effects against influenza A virus and HIV^78–80^. In addition to the anti-viral effect of *H. perforatum* extract, *H. perforatum* ethylacetate (HPE) extract showed a remarkable decrease in the concentration of IL-6 and TNF-α through the NF-κB in lung tissue of mice infected with an influenza A virus^80^ and in the trachea and kidney of chickens infected with IBV, mainly from hypericin content of HPE^17^.

The observation that the highest inhibition was shown upon virus treatment followed by cells treatment before infection indicate the potential role that *H. perforatum* might play in virus-cell interaction (Figs. 2 and 3). *H. perforatum* also showed prolonged activity up to 48 h compared to *H. perforatum- Echinacea* mixture or *Echinacea* alone indicating its potential long-acting effect when used as antiviral against SARS-CoV-2.

Although *Echinacea* has been shown to have antiviral activity by other studies^81–84^, the addition of *Echinacea* to *H. perforatum* resulted in decreasing the antiviral activity. Studies have shown that *Echinacea* has a better effect as a cytokine regulator as shown by others^85,86^. Therefore an in vivo study is needed to evaluate the effect of *Echinacea* on cytokine regulation during COVID-19 infection together with the anti-viral effect found in this study of *H. perforatum*. The in vivo experiments should also investigate the anti-inflammatory effect of the mixture by measuring the mRNA expression levels of pro-inflammatory cytokines such as: IL-6, TNF-α, INF-β. This reduction in the inflammation is expected to empower the viral inhibition effect of the medicinal plants as reported in previous studies^17,20,23,31,34,37,46,48,63,72^.

In silico finding using molecular docking and MD simulation suggests that Echinacin and Echinacoside from *E. angustifolia*, Cyanin and Cyanidin 3-(6ʺ-malonylglucoside) from *E. purpurea*, and Quercetin-3-O-glucuronide and Proanthocyanidins from *H. perforatum* have good inhibitory potential against SARS-CoV-2 M^pro^ via the inhibition of viral M^pro^ proteins (Figs. 6, 8). Likewise, Echinacoside and Rutin from *E. angustifolia*, Kaempferol-3-O-rutinoside and Echinacoside from *E. purpurea*, and Rutin and Quercetin-3-O-xyloside from *H. perforatum* were identified to have good binding with SARS-CoV-2 RdRp (Figs. 6, 8). In hence, as observed in the in vitro assays (Fig. 1), identified phytochemicals from the selected plants were marked as potent inhibitors for main protease and RdRp, which are the essential proteins required in the initiation and replication of SARS-CoV-2 respectively.

Conclusively, Echinacoside and Rutin are considered as multi targeted compounds due to their good in silico inhibitory potential against both viral targets. Therefore, the identified phytomolecules can be considered for further in vitro and in vivo validation for designing new potential drugs against SARS-CoV-2. As the standard treatment of COVID-19 is constantly changing with the new developments of antiviral therapies, future studies should include a comparison of the standard treatment at the time with the results from this study.

## Supplementary Information

Supplementary Information.

## Data Availability

All data generated or analyzed during this study are included in this published article and its supplementary information files.
