# Supplementary material for: In vitro screening of anti-viral and virucidal effects against SARS-CoV-2 by Hypericum perforatum and Echinacea
Source: Sci Rep. 2022 Dec 15;12:21723. doi: 10.1038/s41598-022-26157-3 (PMC9754313; doi:10.1038/s41598-022-26157-3)
Supplement: Supplementary file 1 — Supplementary Information. [file 41598_2022_26157_MOESM1_ESM.docx]

Table S1. Virtual screening of compounds from *E.* angustifolia against SARS-CoV-2 M^pro^.

| **S. no.** | **CID** | **Compound Name** | **Docking Score** |
| --- | --- | --- | --- |
|  | 5281771 | Echinacoside | -10.6 |
|  | 5280805 | Rutoside | -10.0 |
|  | 6439941 | Echinacin | -9.0 |
|  | 5282102 | Kaempferol-3-Glucoside | -8.9 |
|  | 6537500 | Echinacea | -8.7 |
|  | 44259796 | Quercetagetin-7-Glucoside | -8.6 |
|  | 5134221 | Chicoric-Acid | -8.2 |
|  | 5280637 | Luteolin-7-Glucoside | -8.2 |
|  | 440946 | Fructans | -8.1 |
|  | 5281769 | Cynarin | -8.0 |
|  | 5280863 | Kaempferol | -8.0 |
|  | 6438981 | Tussilagin | -8.0 |
|  | 5280794 | Stigmastero | -7.6 |
|  | 5281654 | Isorhamnetin | -7.6 |
|  | 5280343 | Quercetin | -7.5 |
|  | 222284 | Beta-Sitosterol | -7.5 |
|  | 6440397 | Caftaric-Acid | -7.5 |
|  | 5742590 | Sitosterol-3-O-Beta-D-Glucoside | -7.5 |
|  | 5280445 | Luteolin | -7.4 |
|  | 5991 | EO ( Ethinyl Estradiol) | -7.0 |
|  | 5315832 | Isochlorogenic-Acid | -7.0 |
|  | 1794427 | Chlorogenic-Acid | -7.0 |
|  | 14350 | Caryophyllene-Epoxide | -7.0 |
|  | 16219508 | Inulin from Dahlia plant | -6.8 |
|  | 5281515 | Caryophyllene | -6.8 |
|  | 93009 | Bornyl-Acetate | -6.5 |
|  | 5280443 | Apigenin | -6.5 |
|  | 91723653 | Germacrene | -6.3 |
|  | 64685 | Borneol | -6.3 |
|  | 11587035 | Echinamine B | -6.1 |
|  | 149801 | Isotussilagine | -6.0 |
|  | 185071 | Tussilagine | -6.0 |
|  | 81653 | Isobutylamides ( N,N-Dimethylisobutylamine) | -5.1 |
|  | 22199 | Behenic-Acid-Ethyl-Ester | -5.0 |
|  | 247 | Betaine | -4.7 |
|  | 5322026 | Trideca-1-11-Diene-3-5-7-9-Tetrayne | -4.3 |
|  | 528755 | Trideca-1,3-Diene-5,7,9,11-Tetrayne | -3.9 |
|  | 441552 | Tridec-1-Ene-3-5-7-9-11-Pentayne | -3.5 |
|  | 131751109 | Echinacea | -3.2 |
|  | 3931 | Linoleic-Acid | -3.2 |
|  | 593849 | 2,4-Undecadiene-8,10-Diynamide, N-(2-Methylpropyl)-, (2Z,4E) | -2.9 |
|  | 10469 | Cirotic Acid | -2.9 |
|  | 22041880 | Dodeca-2,4-Dien-1-Yl-Isovalerate | -2.7 |
|  | 11413953 | Dodeca-2,4,8,10-Tetraen-1-Oic-Acid-Isobutylamide.1 | -2.6 |

Table S2. Virtual screening of compounds from *E.*angustifolia against SARS-CoV-2 RdRp.

| **S.no.** | **CID** | **Compound name** | **Docking score (kcal/mol)** |
| --- | --- | --- | --- |
|  | 5281771 | Echinacoside | -7.7 |
|  | 6439941 | Echinacin | -6.7 |
|  | 5280805 | Rutoside | -6.6 |
|  | 44259796 | Quercetagetin-7-glucoside | -6.5 |
|  | 5282102 | Kaempferol-glucoside | -5.9 |
|  | 5280637 | Luteolin-7-glucoside | -5.8 |
|  | 6537500 | Echinacea | -5.6 |
|  | 5280863 | Kaempferol | -5.6 |
|  | 5134221 | Chicoric-acid | -5.5 |
|  | 5281769 | Cynarin | -5.3 |
|  | 6440397 | Caftaric-acid | -5.3 |
|  | 5280343 | Quercetin | -5.2 |
|  | 5315832 | Isochlorogenic-acid | -5.2 |
|  | 1794427 | Chlorogenic-acid | -5.2 |
|  | 5281654 | Isorhamnetin | -5.2 |
|  | 16219508 | Inulin | -5.1 |
|  | 440946 | Fructans | -5.1 |
|  | 5280443 | 11 apigenin | -5.0 |
|  | 5280445 | Luteolin | -4.9 |
|  | 5742590 | Sitosterol-3-o-beta-d-glucoside | -4.7 |
|  | 64685 | Borneol | -4.5 |
|  | 6438981 | Tussilagin | -4.4 |
|  | 149801 | Isotussilagine | -3.8 |
|  | 185071 | Tussilagine | -3.8 |
|  | 222284 | Beta-sitosterol | -3.6 |
|  | 5280794 | Stigmasterol | -3.6 |
|  | 14350 | Caryophyllene-epoxide | -3.5 |
|  | 93009 | Bornyl-acetate | -3.4 |
|  | 91723653 | Germacrene | -3.3 |
|  | 247 | Betaine | -3.3 |
|  | 5281515 | Caryophyllene | -3.1 |
|  | 81653 | Isobutylamides ( N,N-dimethylisobutylamine) | -2.9 |
|  | 5322026 | Trideca-1-11-diene-3-5-7-9-tetrayne | -1.1 |
|  | 131751109 | Echinacein | -0.7 |
|  | 593849 | 2,4-Undecadiene-8,10-diynamide, N-(2-methylpropyl)-, (2Z,4E) | -0.3 |
|  | 11413953 | Dodeca-2,4,8,10-tetraen-1-oic-acid-isobutylamide.1 | -0.1 |

Table S3. Virtual screening of compounds from *E. purpurea* against SARS-CoV-2 M^pro^.

| **S. no.** | **CID** | **Compound** | **Docking score (kcal/mol)** |
| --- | --- | --- | --- |
|  | 5281771 | Echinacoside | -10.6 |
|  | 441688 | Cyanin | -10.0 |
|  | 5280805 | Rutin | -10.0 |
|  | 5318767 | Kaempferol-3-0-rutinoside | -10.0 |
|  | 44256740 | Cyanidin-3-o-(6-o-malonyl-beta-d-glucopyranoside) | -9.2 |
|  | 6439941 | Echinacin | -9.0 |
|  | 5281764 | (-)-l-chicoric acid.1 | -8.5 |
|  | 5282160 | Quercetin-7-glucoside | -8.1 |
|  | 6438981 | Tussilagone | -8.0 |
|  | 493570 | Riboflavin | -7.8 |
|  | 5280343 | Quercetin | -7.6 |
|  | 222284 | Beta-sitosterol | -7.5 |
|  | 54608033 | Cinnamoyl-echinadiol | -7.4 |
|  | 24847856 | Arabinogalactan | -7.3 |
|  | 14350 | Caryophyllene-epoxide | -6.9 |
|  | 5281515 | Caryophyllene | -6.8 |
|  | 93009 | Bornyl-acetate | -6.5 |
|  | 1130 | Thiamine | -6.4 |
|  | 91723653 | Germacrene | -6.3 |
|  | 64685 | Borneol | -6.3 |
|  | 1183 | Vanillin | -6.0 |
|  | 185071 | Tussilagine | -6.0 |
|  | 149801 | Isotussilagine | -6.0 |
|  | 938 | Niacin | -5.6 |
|  | 5319562 | P-hydroxycinnamic-acid-methyl-ester | -5.5 |
|  | 637542 | P-coumaric-acid | -5.0 |
|  | 54670067 | Ascorbic-acid | -4.9 |
|  | 247 | Betaine | -4.7 |
|  | 5322026 | Trideca-1-11-diene-3-5-7-9-tetrayne | -4.3 |
|  | 528755 | Trideca-1,3-diene-5,7,9,11-tetrayne | -3.9 |
|  | 441552 | Tridec-1-ene-3-5-7-9-11-pentayne | -3.5 |
|  | 15609884 | 2,4-undecadiene-8,10-diynoic acid isobutylamide | -2.9 |

Table S4. Virtual screening of compounds from *E. purpurea* against SARS-CoV-2 RdRp.

| **S.no.** | **CID** | **Compound** | **Docking score (kcal/mol)** |
| --- | --- | --- | --- |
|  | 5281771 | Echinacoside | -7.7 |
|  | 441688 | Cyanin | -6.8 |
|  | 6439941 | Echinacin | -6.7 |
|  | 5280805 | Rutoside | -6.6 |
|  | 5318767 | Kaempferol-3-0-rutinoside | -6.2 |
|  | 5282160 | Quercetin-7-glucoside | -6.1 |
|  | 44256740 | Cyanidin-3-o-(6-o-malonyl-beta-d-glucopyranoside | -6.0 |
|  | 5281764 | Chichoric-acid | -5.5 |
|  | 5281764 | (-)-l-chicoric acid.1 | -5.4 |
|  | 493570 | Riboflavin | -5.4 |
|  | 5280343 | Quercetin | -5.2 |
|  | 24847856 | Arabinogalactan | -5.1 |
|  | 1183 | Vanillin | -4.6 |
|  | 24206434 | Gum rosin | -4.5 |
|  | 64685 | Borneol | -4.5 |
|  | 54608033 | Cinnamoyl-echinadiol | -4.3 |
|  | 54670067 | Ascorbic-acid | -4.2 |
|  | 938 | Niacin | -4.1 |
|  | 14350 | Caryophyllene-epoxide | -4.0 |
|  | 637542 | P-coumaric-acid | -3.9 |
|  | 149801 | Isotussilagine | -3.8 |
|  | 185071 | Tussilagine | -3.8 |
|  | 6438981 | Tussilagone | -3.6 |
|  | 222284 | Beta-sitosterol | -3.6 |
|  | 5319562 | P-hydroxycinnamic-acid-methyl-ester | -3.5 |
|  | 93009 | Bornyl-acetate | -3.4 |
|  | 91723653 | Germacrene | -3.3 |
|  | 247 | Betaine | -3.3 |
|  | 5281515 | Caryophyllene | -3.1 |
|  | 1130 | Thiamine | -3.1 |
|  | 750 | Glycine | -1.8 |
|  | 5322026 | Trideca-1-11-diene-3-5-7-9-tetrayne | -1.1 |

Table S5. Virtual screening of compounds from *H. perforatum* against SARS-CoV-2 M^pro^.

| **S. No.** | **CID** | **Compound name** | **Docking score (kcal/mol)** |
| --- | --- | --- | --- |
|  | 5280805 | Rutin | -10.2 |
|  | 107876 | Proanthocyanidins | -10.0 |
|  | 5274585 | Quercetin-3-o-glucuronide | -9.2 |
|  | 5280459 | Quercitrin | -8.7 |
|  | 138911134 | Myricetin-3-o-beta-d-glucoside | -8.2 |
|  | 5280804 | Isoquercitrin | -8.2 |
|  | 5281643 | Hyperin | -8.2 |
|  | 10813969 | Isoquercitin | -8.2 |
|  | 5281647 | Mangiferin | -8.2 |
|  | 5280863 | Kaempferol | -8.0 |
|  | 71629 | Leucocyanidin | -7.9 |
|  | 5280343 | Quercetin | -7.9 |
|  | 1203 | Catechins | -7.8 |
|  | 72276 | (-)-epicatechin | -7.8 |
|  | 9064 | (+)-catechin | -7.8 |
|  | 182232 | (+)-epicatechin | -7.8 |
|  | 5320863 | Quercetin-3-o-xyloside | -7.6 |
|  | 128861 | Cyanidin | -7.6 |
|  | 9963735 | Adhyperfolin | -7.6 |
|  | 4978 | Pseudohypericin | -7.5 |
|  | 5280445 | Luteolin | -7.4 |
|  | 10414856 | Biapigenin | -7.4 |
|  | 198016 | Saponin | -7.3 |
|  | 227829 | Guaiol | -7.2 |
|  | 91457 | Beta-eudesmol | -7.2 |
|  | 222284 | Beta-sitosterol | -7.2 |
|  | 12303662 | Phytosterols | -7.2 |
|  | 62566 | Beta-bourbonene | -7.0 |
|  | 5281600 | Amentoflavone | -7.0 |
|  | 14350 | Caryophyllene-epoxide | -7.0 |
|  | 1794427 | Chlorogenic-acid | -7.0 |
|  | 441298 | Hyperforin | -6.9 |
|  | 13834128 | Kielcorin | -6.9 |
|  | 122635 | Emodinanthranol | -6.9 |
|  | 92762 | Alpha-eudesmol | -6.8 |
|  | 15825655 | 2,2-dimethyl-7-sec-butyl-2h,5h-pyrano-(4,3-b)-pyran-5-one | -6.8 |
|  | 136161635 | Isohypericin | -6.8 |
|  | 12306052 | Alpha-amorphene | -6.7 |
|  | 5281515 | Caryophyllene | -6.7 |
|  | 3032853 | Cadinene | -6.7 |
|  | 5281672 | Myricetin | -6.7 |
|  | 15406 | Fenchol | -6.6 |
|  | 441005 | Delta-cadinene | -6.6 |
|  | 5281426 | Umbelliferone | -6.6 |
|  | 5281656 | 1,3,6,7-tetrahydroxyxanthone | -6.6 |
|  | 10856614 | Alpha-selinene | -6.6 |
|  | 6432005 | Gamma-eudesmol | -6.5 |
|  | 6448 | Bornyl-acetate | -6.5 |
|  | 135159464 | Cyclopseudohypericin | -6.5 |
|  | 14619932 | Ishwarane | -6.5 |
|  | 73145 | Beta-amyrin | -6.5 |
|  | 442393 | Beta-selinene | -6.5 |
|  | 15560275 | Gurjunene | -6.4 |
|  | 15825656 | 2,2-dimethyl-7-isobutyl-2h,5h-pyrano-(4,3-b)-pyran-5-one | -6.4 |
|  | 5316205 | Alpha-cuprenene | -6.3 |
|  | 64685 | Borneol | -6.3 |
|  | 115250 | Taraxasterol | -6.3 |
|  | 5280460 | Scopoletin | -6.2 |
|  | 171335 | Protopseudohypericin | -6.2 |
|  | 2758 | Cineole | -6.2 |
|  | 14896 | Beta-pinene | -6.2 |
|  | 7460 | Alpha-phellandrene | -6.1 |
|  | 92138 | Elemol | -6.1 |
|  | 5281520 | Humulene | -6.1 |
|  | 6616 | Camphene | -6.1 |
|  | 11230 | Terpinen-4-ol | -6.1 |
|  | 6654 | Alpha-pinene | -6.1 |
|  | 15837102 | Pinene | -6.0 |
|  | 7463 | P-cymene | -6.0 |
|  | 7410 | Acetophenone | -6.0 |
|  | 164660 | Protohypericin | -6.0 |
|  | 7461 | Gamma-terpinene | -6.0 |
|  | 101153 | Pinol | -6.0 |
|  | 6427714 | Selina-4,11-diene | -5.9 |
|  | 135 | P-hydroxy-benzoic-acid | -5.9 |
|  | 7462 | Alpha-terpinene | -5.9 |
|  | 111037 | Alpha-terpinyl-acetate | -5.9 |
|  | 92139 | Alpha-Curcumene | -5.9 |
|  | 3469 | Gentisic-acid | -5.8 |
|  | 359 | Phloroglucinol | -5.8 |
|  | 12304273 | Gamma-curcumene | -5.7 |
|  | 938 | Nicotinic-acid | -5.6 |
|  | 6918391 | Beta-elemene | -5.6 |
|  | 370 | Gallic-acid | -5.6 |
|  | 56842786 | Bicycloelemene | -5.5 |
|  | 8468 | Vanillic-acid | -5.5 |
|  | 76336 | Rhodan | -5.5 |
|  | 17100 | Alpha-terpineol | -5.5 |
|  | 736186 | Isoferulic-acid | -5.5 |
|  | 445858 | Ferulic-acid | -5.4 |
|  | 61284 | Alpha-campholenol | -5.4 |
|  | 1057 | Pyrogallol | -5.3 |
|  | 22311 | Limonene | -5.3 |
|  | 3663 | Hypericin | -5.2 |
|  | 689043 | Caffeic-acid | -5.2 |
|  | 54670067 | Ascorbic-acid | -5.1 |
|  | 5365910 | Methyl-geranate | -5.1 |
|  | 85334092 | Acylphloroglucinols | -5.0 |
|  | 145742 | Proline | -5.0 |
|  | 637542 | P-coumaric-acid | -5.0 |
|  | 444539 | Cinnamic-acid | -4.8 |
|  | 1549026 | Geranyl-acetate | -4.8 |
|  | 1549025 | Neryl-acetate | -4.7 |
|  | 10430 | Isovalerianic-acid | -4.5 |
|  | 6549 | Linalool | -4.5 |
|  | 637566 | Geraniol | -4.2 |
|  | 643820 | Nerol | -4.2 |
|  | 6262 | Ornithine | -4.1 |
|  | 6251 | Mannitol | -4.0 |
|  | 6288 | Threonine | -3.9 |
|  | 638011 | Geranial | -3.9 |
|  | 643779 | Neral | -3.8 |
|  | 31253 | Myrcene | -3.0 |
|  | 11005 | Myristic-acid | -1.4 |

Table S6. Virtual screening of compounds from *H. perforatum* against SARS-CoV-2 RdRp.

| **S.no.** | **PubChem CID** | **Compound name** | **Docking score** |
| --- | --- | --- | --- |
|  | 5280805 | Rutin | -6.9 |
|  | 107876 | Proanthocyanidins | -6.6 |
|  | 71629 | Leucocyanidin | -6.0 |
|  | 128861 | Cyanidin | -6.0 |
|  | 5320863 | Quercetin-3-o-xyloside | -6.0 |
|  | 1203 | Catechins | -5.9 |
|  | 72276 | (-)-Epicatechin | -5.9 |
|  | 9064 | (+)-Catechin | -5.9 |
|  | 182232 | (+)-Epicatechin | -5.9 |
|  | 5280459 | Quercitrin | -5.9 |
|  | 5274585 | Quercetin-3-o-glucuronide | -5.9 |
|  | 1794427 | Chlorogenic-acid | -5.8 |
|  | 359 | Phloroglucinol | -5.8 |
|  | 138911134 | Myricetin-3-o-beta-d-glucoside | -5.7 |
|  | 5281647 | Mangiferin | -5.7 |
|  | 5281672 | Myricetin | -5.7 |
|  | 135 | P-hydroxy-benzoic-acid | -5.7 |
|  | 5280804 | Isoquercitrin | -5.7 |
|  | 5281643 | Hyperin | -5.7 |
|  | 10813969 | Isoquercitin | -5.7 |
|  | 5280863 | Kaempferol | -5.6 |
|  | 5280343 | Quercetin | -5.5 |
|  | 370 | Gallic-acid | -5.5 |
|  | 3469 | Gentisic-acid | -5.5 |
|  | 85334092 | Acylphloroglucinols | -5.3 |
|  | 8468 | Vanillic-acid | -5.1 |
|  | 5280445 | Luteolin | -5.0 |
|  | 54670067 | Ascorbic-acid | -4.8 |
|  | 122635 | Emodinanthranol | -4.8 |
|  | 15406 | Fenchol | -4.8 |
|  | 10414856 | Biapigenin | -4.7 |
|  | 5281656 | 1,3,6,7-tetrahydroxyxanthone | -4.7 |
|  | 5281426 | Umbelliferone | -4.7 |
|  | 1057 | Pyrogallol | -4.7 |
|  | 13834128 | Kielcorin | -4.6 |
|  | 5280460 | Scopoletin | -4.5 |
|  | 5281600 | Amentoflavone | -4.5 |
|  | 6288 | Threonine | -4.5 |
|  | 64685 | Borneol | -4.5 |
|  | 227829 | Guaiol | -4.4 |
|  | 736186 | Isoferulic-acid | -4.4 |
|  | 6616 | Camphene | -4.4 |
|  | 17100 | Alpha-terpineol | -4.4 |
|  | 11230 | Terpinen-4-ol | -4.3 |
|  | 445858 | Ferulic-acid | -4.2 |
|  | 6432005 | Gamma-eudesmol | -4.2 |
|  | 938 | Nicotinic-acid | -4.1 |
|  | 6654 | Alpha-pinene | -4.1 |
|  | 91457 | Beta-eudesmol | -4.1 |
|  | 10430 | Isovalerianic-acid | -4.1 |
|  | 6262 | Ornithine | -4.1 |
|  | 145742 | Proline | -4.0 |
|  | 15837102 | Pinene | -4.0 |
|  | 7410 | Acetophenone | -4.0 |
|  | 14896 | Beta-pinene | -3.9 |
|  | 637542 | P-coumaric-acid | -3.9 |
|  | 14350 | Caryophyllene-epoxide | -3.9 |
|  | 3032853 | Cadinene | -3.9 |
|  | 689043 | Caffeic-acid | -3.9 |
|  | 222284 | Beta-sitosterol | -3.8 |
|  | 12303662 | Phytosterols | -3.8 |
|  | 92762 | Alpha-eudesmol | -3.7 |
|  | 6251 | Mannitol | -3.6 |
|  | 6448 | Bornyl-acetate | -3.6 |
|  | 115250 | Taraxasterol | -3.6 |
|  | 7461 | Gamma-terpinene | -3.6 |
|  | 62566 | Beta-bourbonene | -3.5 |
|  | 7460 | Alpha-phellandrene | -3.5 |
|  | 7463 | P-cymene | -3.5 |
|  | 442393 | Beta-selinene | -3.5 |
|  | 73145 | Beta-amyrin | -3.5 |
|  | 441005 | Delta-cadinene | -3.5 |
|  | 12306052 | Alpha-amorphene | -3.4 |
|  | 7462 | Alpha-terpinene | -3.4 |
|  | 92138 | Elemol | -3.4 |
|  | 171335 | Protopseudohypericin | -3.4 |
|  | 76336 | Rhodan | -3.4 |
|  | 15560275 | Gurjunene | -3.4 |
|  | 10856614 | Alpha-selinene | -3.3 |
|  | 111037 | Alpha-terpinyl-acetate | -3.2 |
|  | 5281520 | Humulene | -3.2 |
|  | 14619932 | Ishwarane | -3.2 |
|  | 5281515 | Caryophyllene | -3.2 |
|  | 9963735 | Adhyperfolin | -3.2 |
|  | 15825655 | 2,2-dimethyl-7-sec-butyl-2h,5h-pyrano-(4,3-b)-pyran-5-one | -3.1 |
|  | 5316205 | Alpha-cuprenene | -3.1 |
|  | 61284 | Alpha-campholenol | -3.1 |
|  | 441298 | Hyperforin | -3.0 |
|  | 444539 | Cinnamic-acid | -2.9 |
|  | 1549026 | Geranyl-acetate | -2.9 |
|  | 15825656 | 2,2-dimethyl-7-isobutyl-2h,5h-pyrano-(4,3-b)-pyran-5-one | -2.8 |
|  | 22311 | Limonene | -2.8 |
|  | 6918391 | Beta-elemene | -2.8 |
|  | 1549025 | Neryl-acetate | -2.7 |
|  | 56842786 | Bicycloelemene | -2.7 |
|  | 643820 | Nerol | -2.6 |
|  | 6427714 | Selina-4,11-diene | -2.5 |
|  | 164660 | Protohypericin | -2.5 |
|  | 5365910 | Methyl-geranate | -2.3 |
|  | 92139 | Ar-curcumene | -2.2 |
|  | 637566 | Geraniol | -2.1 |
|  | 6549 | Linalool | -2.1 |
|  | 643779 | Neral | -1.8 |
|  | 638011 | Geranial | -1.6 |

Table S7. Re-docking analysis of top ten compounds from *E. angustifolia* against SARS-CoV-2 M^pro^.

| **S. no.** | **Compound** | **CID** | **Docking score (kcal/mol)** |
| --- | --- | --- | --- |
|  | Echinacin | 6439941 | -9 |
|  | Echinacoside | 5281771 | -8.6 |
|  | Astragalin | 5282102 | -8.8 |
|  | Cynarin (Echinacea)(1,3-Dicaffeoylquinic Acid) | 6537500 | -8.8 |
|  | Quercetagetin 7-glucoside | 44259796 | -8.7 |
|  | Cynaroside | 5280637 | -8.3 |
|  | Rutin | 5280805 | -8.2 |
|  | Cichoric acid | 5134221 | -8.2 |
|  | Levan n | 440946 | -7.4 |
|  | Cynarin | 5281769 | -7.3 |

Table S8. Re-docking analysis of top ten compounds from *E. angustifolia* against SARS-CoV-2 RdRp.

| **S.no.** | **Compound name** | **CID** | **Docking score (kcal/mol)** |
| --- | --- | --- | --- |
|  | Echinacoside | 5281771 | -9.2 |
|  | Rutin | 5280805 | -9.0 |
|  | Cynarin (echinacea)(1,3-dicaffeoylquinic acid) | 6537500 | -8.4 |
|  | Cynarin | 5281769 | -8.1 |
|  | Astragalin | 5282102 | -8 |
|  | Cynaroside | 5280637 | -8 |
|  | Echinacin | 6439941 | -7.9 |
|  | Quercetagetin 7-glucoside | 44259796 | -7.7 |
|  | Cichoric acid | 5134221 | -7.6 |
|  | Kaempferol | 5280863 | -7.6 |

Table S9. Re-docking analysis of top ten compounds from *E. purpurea* against SARS-CoV-2 M^pro^.

| **S.no.** | **Compound** | **CID** | **Docking score (kcal/mol)** |
| --- | --- | --- | --- |
|  | Cyanin | 441688 | -9.6 |
|  | Cyanidin 3-(6''-malonylglucoside) | 44256740 | -9 |
|  | Echinacin | 6439941 | -9 |
|  | Quercimeritrin | 5282160 | -9 |
|  | Kaempferol-3-O-rutinoside | 5318767 | -8.8 |
|  | Echinacoside | 5281771 | -8.6 |
|  | RUTIN | 5280805 | -8.2 |
|  | (-)-L-Chicoric acid | 5281764 | -8.1 |
|  | Chicoric acid | 5281764 | -8 |
|  | Tussilagone | 6438981 | -6.6 |

Table S10. Re-docking analysis of top ten compounds from *E. purpurea* against SARS-CoV-2 RdRp.

| **S.no.** | **Compound** | **CID** | **Docking score (kcal/mol)** |
| --- | --- | --- | --- |
|  | Kaempferol-3-o-rutinoside | 5318767 | -9.3 |
|  | Echinacoside | 5281771 | -9.2 |
|  | Rutoside | 5280805 | -9.2 |
|  | Cyanin | 441688 | -8.4 |
|  | Quercetin-7-glucoside | 5282160 | -8.1 |
|  | Cyanidin-3-o-(6-o-malonyl-beta-d-glucopyranoside) | 44256740 | -8.1 |
|  | Echinacin | 6439941 | -7.9 |
|  | (-)-l-chicoric acid | 5281764 | -7.7 |
|  | Cichoric acid | 5134221 | -7.5 |
|  | Riboflavin | 493570 | -6.7 |

Table S11: Re-docking analysis of top ten compounds from *H. perforatum* against SARS-CoV-2 M^pro^.

| **S. no.** | **Compound** | **CID** | **Docking score (kcal/mol)** |
| --- | --- | --- | --- |
|  | Quercetin-3-O-glucuronide | 5274585 | -9.6 |
|  | Proanthocyanidins | 107876 | -9.1 |
|  | Rutin | 5280805 | -8.8 |
|  | Myricetin-3-o-beta-d-glucoside | 138911134 | -8.8 |
|  | Quercitrin | 5280459 | -8.5 |
|  | Mangiferin | 5281647 | -8.4 |
|  | Isoquercitrin | 5280804 | -8.3 |
|  | Hyperin | 5281643 | -8.3 |
|  | Isoquercitin | 10813969 | -8.3 |
|  | Kaempferol | 5280863 | -7.4 |

Table S12: Re-docking analysis of top ten compounds from *H. perforatum* against SARS-CoV-2 RdRp.

| **S. no.** | **Compound** | **CID** | **Docking score (kcal/mol)** |
| --- | --- | --- | --- |
|  | Rutin | 5280805 | -9.0 |
|  | Quercetin-3-o-xyloside | 5320863 | -8 |
|  | Proanthocyanidins | 107876 | -7.7 |
|  | (-)-epicatechin | 72276 | -7.7 |
|  | Quercitrin | 5280459 | -7.6 |
|  | Catechins | 1203 | -7.5 |
|  | (+)-catechin | 9064 | -7.5 |
|  | (+)-epicatechin | 182232 | -7.5 |
|  | Cyanidin | 128861 | -7.4 |
|  | Leucocyanidin | 71629 | -6.9 |

Table S13. Inhibitory effects of H. perforatum, Echinacea and H. perforatum-Echinacea mixture on SARS-CoV-2 through infection in Vero E6 cells.

| Plant  materials | Post-Treatment of Virus-infected Cells (Anti-viral Activity) | | | | |
| --- | --- | --- | --- | --- | --- |
|  | Viral Inhibition (%) | | | | |
|  | **12h** | **16h** | **24h** | **36h** | **48h** |
| *H. perforatum* | 0.64 | 1.45 | 5.54 | 17.75 | 13.76 |
| *Echinacea* | 0.0 | 0.0 | 0.0 | 0.0 | 0.0 |
| Mixture | 0.0 | 2.52 | 2.09 | 6.02 | 6.51 |
| Plant  materials | Pre-treatment of Cells prior to Virus Infection (Anti-viral Activity) | | | | |
|  | Viral Inhibition (%) | | | | |
|  | **12h** | **16h** | **24h** | **36h** | **48h** |
| *H. perforatum* | 5.32 | 5.96 | 17.67 | 29.64 | 25.22 |
| *Echinacea* | 0.36 | 0.18 | 4.52 | 1.53 | 3.29 |
| Mixture | 4.84 | 2.86 | 10.84 | 16.11 | 22.58 |
| Plant  materials | Pre-treatment of Virus Prior to Infection (Virucidal Activity) | | | | |
|  | Viral Inhibition (%) | | | | |
|  | **12h** | **16h** | **24h** | **36h** | **48h** |
| *H. perforatum* | 4.41 | 9.93 | 18.62 | 35.33 | 35.77 |
| *Echinacea* | 3.56 | 4.34 | 9.04 | 3.30 | 4.50 |
| Mixture | 5.86 | 8.75 | 19.06 | 31.36 | 27.07 |

**Figure S1**. Cytotoxic effect of the used extracts on Vero E6 and HEK293 cells as determined by MTT assay. The different concentrations of *H. perforatum, Echinacea* and *H. perforatum-Echinacea* mixture ranged from 0.039 to 5 µg/mL. The cytotoxic effect of the cells was calculated relative to cell control wells.

**Figure S2**. Antiviral response curve for *H. perforatum, Echinacea* and the *H. perforatum-Echinacea* mixture activities. The curves represent the % inhibition curve for SARS-CoV-2 infection (Y-axis) against log concentration (µg/mL) of the used extracts (X-axis).


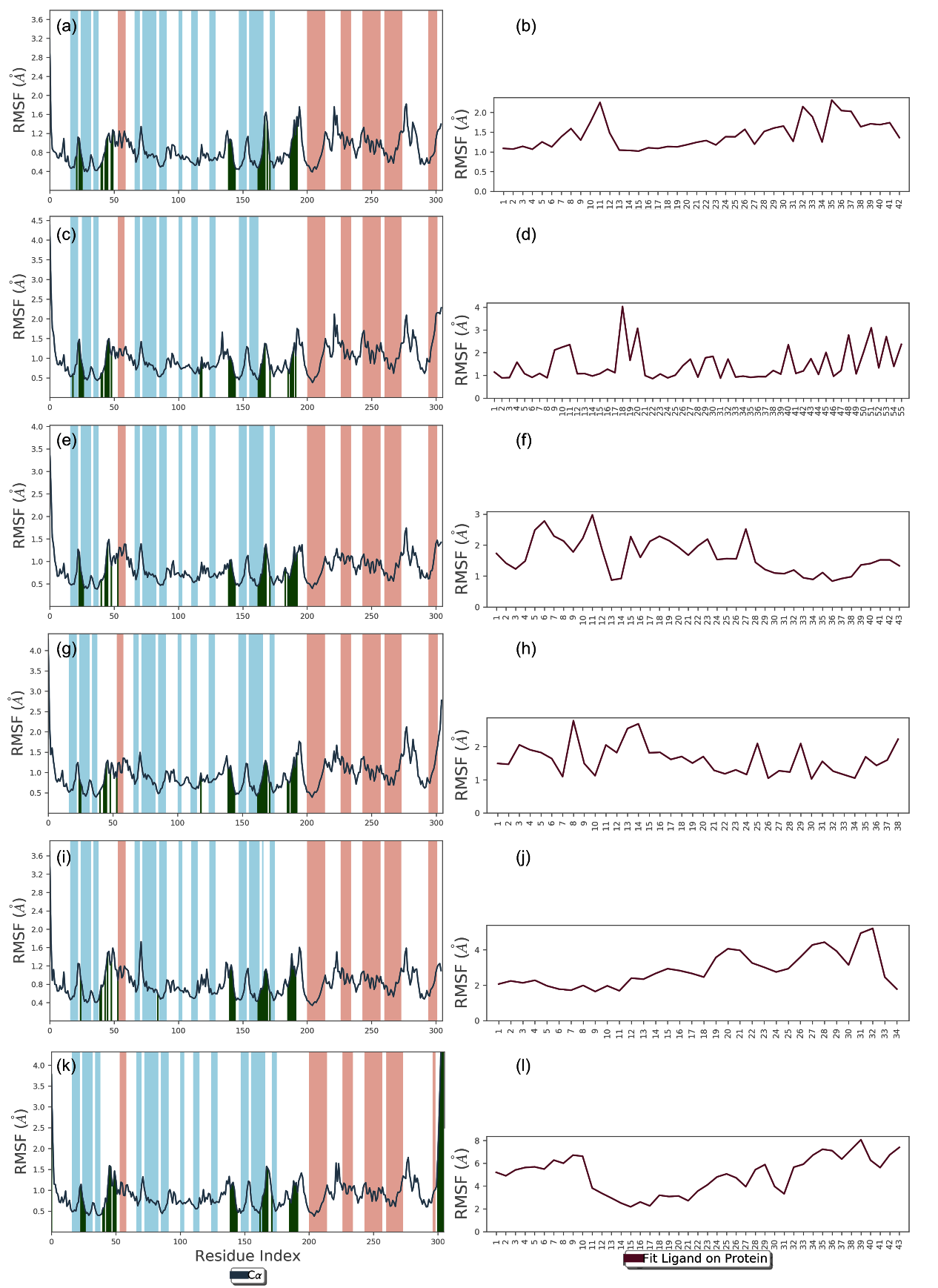


**Figure S3**: Calculated RMSF values for alpha carbon (Cα) atoms (blue curves and) of SARS-CoV-2 proteins and docked ligands (red curves), viz. (a-b) SARS-CoV-2 M^pro^-Echinacin, (c-d) SARS-CoV-2 M^pro^-Echinacoside, (e-f) SARS-CoV-2 M^pro^-Cyanin, (g-h) SARS-CoV-2 M^pro^-Cyanidin 3-(6''-alonylglucoside), (i-j) SARS-CoV-2 M^pro^-Quercetin-3-O-glucuronide, and (k-l) SARS-CoV-2 M^pro^-Proanthocyanidins. Herein, vertical green lines stand for the ligand contacts with residues of the protein, blue and red bands represent the beta and alpha helix secondary structures in the protein.


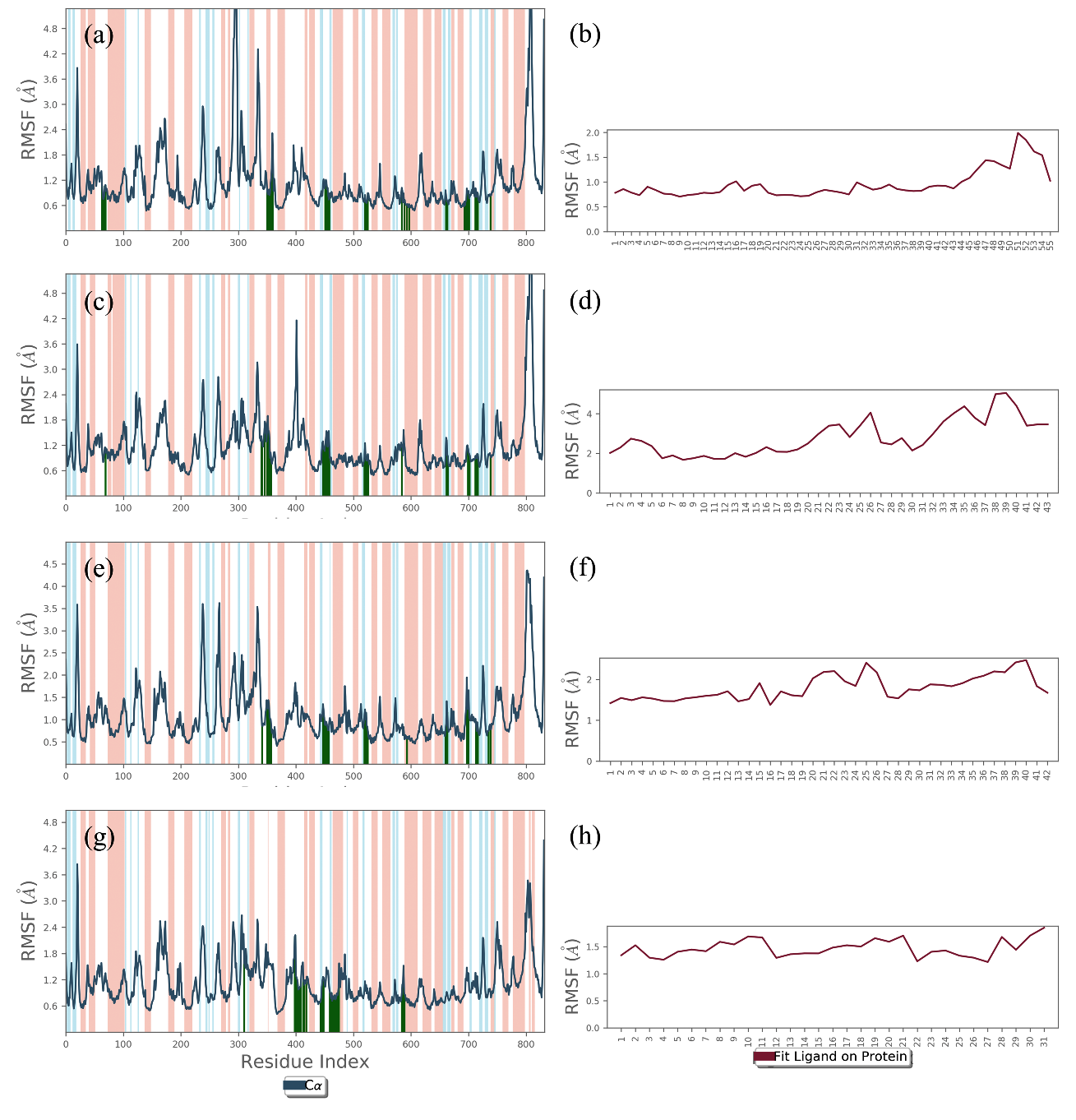


**Figure S4**: Calculated RMSF values for alpha carbon (Cα) atoms (blue curves and) of SARS-CoV-2 proteins and docked ligands (red curves), viz. (a-b) SARS-C0V-2 RdRp-Echinacoside, (c-d) SARS-CoV-2 RdRp-Rutin, (e-f) SARS-C0V-2 RdRp-Kaempferol-3-O-rutinoside, and (g-h) SARS-CoV-2 RdRp-Quercetin-3-O-xyloside, were plotted with respect to 100 ns simulation interval.


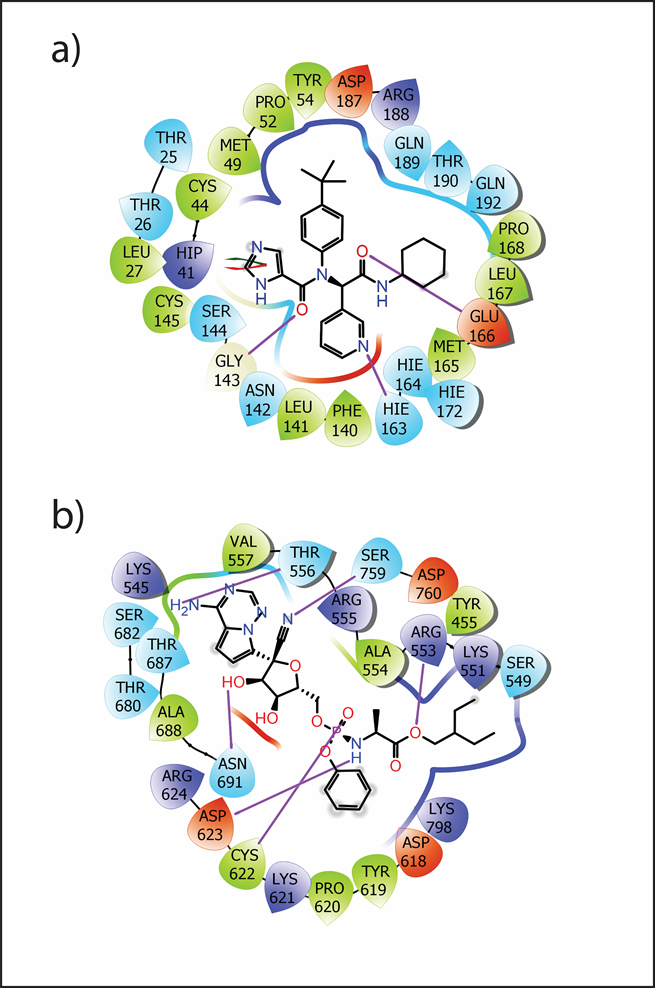


**Figure S5**: 2D interaction diagrams of the target protein and control ligand: (a) SARS-CoV-2 Mpro -X77 complex. (b) SARS-CoV-2 RdRp-Remdesivir complex
